# Remodeling of anti-tumor immunity with antibodies targeting a p53 mutant

**DOI:** 10.1186/s13045-024-01566-1

**Published:** 2024-06-18

**Authors:** Dafei Chai, Junhao Wang, Chunmei Fan, Jing-Ming Lim, Xu Wang, Praveen Neeli, Xinfang Yu, Ken H. Young, Yong Li

**Affiliations:** 1 Department of Medicine, Section of Epidemiology and Population Sciences, Dan L Duncan Comprehensive Cancer Center, Baylor College of Medicine, One Baylor Plaza, Houston, TX USA; 2 Department of Pathology, Division of Hematopathology, Duke University Medical Center, Durham, NC USA

**Keywords:** Mutant p53, E285K, Monoclonal antibody, IgG1, dIgA

## Abstract

**Background:**

p53, the most frequently mutated gene in cancer, lacks effective targeted drugs.

**Methods:**

We developed monoclonal antibodies (mAbs) that target a p53 hotspot mutation E285K without cross-reactivity with wild-type p53. They were delivered using lipid nanoparticles (LNPs) that encapsulate DNA plasmids. Western blot, BLI, flow cytometry, single-cell sequencing (scRNA-seq), and other methods were employed to assess the function of mAbs in vitro and in vivo.

**Results:**

These LNP-pE285K-mAbs in the IgG1 format exhibited a robust anti-tumor effect, facilitating the infiltration of immune cells, including CD8^+^ T, B, and NK cells. scRNA-seq revealed that IgG1 reduces immune inhibitory signaling, increases MHC signaling from B cells to CD8^+^ T cells, and enriches anti-tumor T cell and B cell receptor profiles. The E285K-mAbs were also produced in the dimeric IgA (dIgA) format, whose anti-tumor activity depended on the polymeric immunoglobulin receptor (PIGR), a membrane Ig receptor, whereas that of IgG1 relied on TRIM21, an intracellular IgG receptor.

**Conclusions:**

Targeting specific mutant epitopes using DNA-encoded and LNP-delivered mAbs represents a potential precision medicine strategy against p53 mutants in TRIM21- or PIGR-positive cancers.

**Supplementary Information:**

The online version contains supplementary material available at 10.1186/s13045-024-01566-1.

## Background

Approximately half of human cancers carry mutations in the p53 tumor suppressor gene [1], contributing to tumor immune escape and facilitating tumor recurrence and metastasis [2, 3]. Researchers have devised various therapeutic approaches targeting mutant p53, including small molecular compounds [4, 5], CRISPR/Cas9 gene therapy [6, 7], small peptides [8, 9], and immunotherapies [10, 11]. The overarching goal is to eliminate mutant p53 expression or restore the functional integrity of wild-type p53 (WT p53) in tumor cells [12, 13]. Despite significant strides in these endeavors, the efficacy of these therapeutic interventions has proven unsatisfactory in clinical trials, and there is no FDA-approved drug targeting mutant p53 [14].

Targeting intracellular oncoproteins such as p53 mutants using monoclonal antibodies (mAbs) is a promising approach. However, this therapeutic approach is challenged by the large size of mAbs and their presumed inability to traverse the cell membrane. Researchers have explored various strategies to overcome this obstacle by using smaller antibody fragments, conjugate antibodies with cell-penetrating peptides (CPPs), nanoparticle delivery systems, antibody-drug conjugates (ADCs), and intrabodies [15–17]. Recently, Conejo-Garcia et al. demonstrated that dimeric immunoglobin A (dIgA) against Kras^G12D^ promotes its expulsion from the cytoplasm and restricts tumor growth. dIgA binds to the IgA/IgM polymeric immunoglobin receptor (PIGR), a membrane protein expressed on the basolateral surface of mucosal epithelia. Recognition of dIgA by PIGR leads to transcytosis, allowing the PIGR-dIgA complex to enter the tumor cells, where Kras^G12D^ is neutralized and expelled. This report supports the development of mutation-specific mAbs as therapeutic agents against PIGR-positive cancers.

mAbs directed against p53 mutations have been developed; for example, mAbs such as PAb240 target the conformation of mutant p53 [18, 19]. However, these mAbs often display cross-reactivity with WT p53, limiting their therapeutic potential [20]. In our previous study, we used a mAb in the IgG1 format directed against p53^R175H^ to treat mice carrying a tumor with mutant p53. We found that the mAbs enriched in the tumors following intramuscular injection with electroporation of DNA plasmids expressing the heavy chain (HC) and light chain (LC), respectively [21]. p53^R175H^ mAbs were detected in tumor cells and the tumor microenvironment (TME, including CD45^+^ immune cells). In this work, we developed mAbs against p53^E285K^ for cancer therapy. E285K is a hotspot p53 mutation that resides within the DNA-binding domain of the p53 protein [22], destabilizes the protein structure, and causes a temperature-dependent reduction in transcriptional activation by p53 [23, 24]. p53^E285K^ induces resistance to apoptosis, G1 arrest failure, a decrease in genomic stability, and promotes tumorigenesis [25, 26]. p53^E285K^ is a recurrent mutation in multiple cancers, including bladder urothelial carcinoma, breast-invasive ductal carcinoma, lung adenocarcinoma, and colon adenocarcinoma [27]. Approximately 40% of p53^E285K^ mutations are found in bladder urothelial carcinoma [28].

We identified an anti-p53^E285K^ mAb with high specificity and affinity. When encoded by DNA plasmids and encapsulated in lipid nanoparticles (LNPs), mAbs, either in IgG1 or dIgA format, can be efficiently delivered into cells and tumors, inhibiting the growth of xenograft tumors by activating several immune cell types. These findings underscore the potential therapeutic impact of targeting the p53^E285K^ mutation using mAbs and highlight the promising outcomes of LNP-mediated DNA medicine in cancer therapy.

## Materials and methods

### Mice and cell lines

C57BL/6J, BALB/c, NOD.Cg-Prkdc (scid) Il2rg (tm1Wjl)/SzJ (NSG) (female, aged 6–8 weeks) and humanized CD34^+^ NSG (Hu-NSG-CD34, female, aged 16 weeks) mice were purchased from Jackson Laboratory (Bar Harbor, ME, USA) and the Center for Comparative Medicine (CCM) of Baylor College of Medicine. The mice were housed in the animal facilities of the CCM under pathogen-free conditions. All procedures were performed with the approval of the Institutional Animal Care and Use Committee (IACUC) of Baylor College of Medicine.

HEK293T (Human embryonic kidney cells), RPMI-8226 (Human plasmacytoma cells with p53 gene harboring the E285K mutation), and BT-474 (Human breast ductal carcinoma cells with p53 gene harboring the E285K mutation) were purchased from the American Type Culture Collection (ATCC). The Expi293 cell line was obtained from Thermo Fisher Scientific. The mouse colon cancer MC38-p53^KO/E285K^ cell line, characterized by stable overexpression of human p53^E285K^ after endogenous p53 knockout (p53^KO^), was generated by knocking out the endogenous p53 mutant alleles (G242V & S238I) and introducing the human p53 gene with the E285K mutation. Expi293 cells, HEK293T, MC38, MC38^KO^, and MC38-p53^KO/E285K^ were cultured as described previously [21]. RPMI-8226 cells were cultured in RPMI-1640 (Gibco) supplemented with 10% FBS and 1×anti-anti solution. BT-474 cells were cultured in Hybri-Care Medium (ATCC, 46-X) supplemented with 1.5 g/L sodium bicarbonate, 10% FBS, and 1×Antibiotic-Antimycotic solution (Gibco).

### Antibody construction

Mouse antibodies against p53^E285K^ were prepared using a previously reported method [18, 19] to obtain hybridomas. Mouse mAbs from hybridoma clones were sequenced by Syd Labs, Inc. (Hopkinton, MA) to obtain sequences coding for the heavy-chain (HL) and light-chain (LC). The HC and LC of the mouse mAbs were fused to the Fc of human IgG1 to the E285K-mAb IgG1. The expression cassettes were cloned into either pTwist (Twist Bioscience, South San Francisco, CA, USA) or gWIZ (Aldevron, Fargo, ND, USA) mammalian expression vectors. Three gWIZ constructs were made for dIgA: i.e. VH sequences, followed by mouse IgA constant region sequence, VL sequence along with mouse kappa light chain constant region sequences, and mouse J-chain (JC) sequence. The Fab region of dIgA is identical to that of IgG1 E285K-mAb. All plasmids were purified from DH5α cells, utilizing an endotoxin-free ZymoPURE™ II Plasmid Maxiprep Kit (Zymo Research, Irvine, CA, USA).

### Antibody expression and purification

Antibody expression was performed using the ExpiFectamineTM 293 Transfection Kit (Gibco). For antibody purification, a NAb Protein G Spin Column Kit (Thermo Scientific, Waltham, MA, USA) was used according to the manufacturer’s protocol. The purified samples were dialyzed against PBS overnight at 4 ℃ using a Slide-A-Lyzer Dialysis Cassette (Thermo Fisher Scientific). Subsequent analysis involved SDS-PAGE, and quantification was conducted on the samples using the Pierce BCA Protein Assay Kit (Thermo Scientific).

### Antibody affinity detection using BLI

The interaction and specificity of His-tagged TrxA-E285K peptides and E285K-mAbs were assessed through Bio-Layer Interferometry (BLI) (Gator Bio, Palo Alto, CA, USA). Both antigens and antibodies were exchanged into Q Buffer (PBS with 0.02% Tween-20, 0.2% BSA, and 0.05% NaN3, pH 7.4). The TrxA-E285K-peptide (analyte), TrxA-R179H peptide (negative control), and purified E285K-mAb were diluted in Q buffer and loaded onto the Ni-NTA sensor chip. To initiate the experiment, the sensor was hydrated in 200 µL Q buffer for 10 min and then exposed to 250 µL Q buffer to establish an initial baseline reading. The sensor was then immersed with the E285K-mAbs for 120 s. After loading, the sensor was exposed to 200 µL antigen for 30 s to obtain another baseline measurement, followed by 120 s exposure to record an association curve. Finally, the sensor was exposed to 250 µL Q buffer to measure dissociation. Following each cycle, the sensor was regenerated using a Gly-HCl (pH 1.5) regeneration buffer. The collected data were reference-subtracted and fitted to a 1:1 binding model (Rmax global fit) using Gator Data Analysis Software (Gator Bio).

### Western blot

Western blotting was performed using the primary antibody E285K-mAb, anti-WT p53-mAb (DO-1, Santa Cruz Biotechnology, sc-126) or anti-TRIM2-mAb (Abcam, ab207728) at 4 °C overnight. This was succeeded by the secondary anti-mouse or anti-rabbit IgG HRP-conjugated antibodies (Cell Signaling Technology, 7076 V, 7074 S). The visualization was done using ECL on Chemidoc (Biorad).

### ELISA

Plates were coated with p53-E285K antigen dissolved in coated buffer (R&D Systems, Minneapolis, MN, USA) overnight at 4 °C. The plates were washed with PBST (pH 7.4) containing 0.05% (v/v) Tween-20 and then blocked with 3% BSA for 1 h. The sample was then added and incubated at room temperature for 2 h. Binding was detected using an HRP-conjugated secondary antibody (Cell Signaling Technology). The reaction was developed using a TMB substrate (R&D Systems) and stopped with 2 N H_2_SO_4_. Finally, absorbance was measured at 450 nm using a plate reader (CLARIOstar, BMG Labtech, USA).

### Nucleic acid encapsulation

LNPs were formulated using a NanoAssemblr Spark Formulation Device (Precision Nanosystems). Briefly, antibody-expressing plasmids (40 µg IgG1 containing HC and LC in a ratio of 1:1; 40 µg dIgA containing HC, LC, and JC in a ratio of 1:1:1) or siRNA (Trim21 siRNA, Thermo Fisher Scientific, ID# 150,993; Pigr siRNA, Thermo Fisher Scientific, ID# 151,101; Control siRNA, Thermo Fisher Scientific, AM4635) were diluted in 80 µl of 25 mM citrate buffer, pH 3.5 (bioWORLD, 40320053-1). Cationic lipid SM-102 (Cayman Chemical, 33,474), Cholesterol (Sigma-Aldrich, C3045), Phospholipid DSPC (Sigma-Aldrich Lipids, P1138) and Pegylated lipid DMG-PEG 2000 (Avanti Polar Lipids, 880,151) were diluted in 100% ethanol (Fisher Scientific, A4094) at molar ratio 51/38/8/3 in 40 µl. The lipids were dissolved in ethanol, and two volumes of nucleic acids were added to the buffer. Both phases were loaded into the NanoAssemblr Spark Cartridge (Precision Nanosystems, NIS0013) with a cap, and microfluidic mixing was performed using the recommended setting no. 9. The lipid products were subsequently purified by dialyzing against Ca^2+^ and Mg^2+^ free PBS at pH 7.4 using a Pur-A-Lyzer Maxi Dialysis Kit (0.1–3 mL, MWCO 6–8 kDa, Sigma-Aldrich, PURX60005) overnight and concentrated by 50 KDa Amicon Ultra-0.5 mL Centrifugal Filters (Merck Millipore, UFC505024) to a final pDNA concentration of 0.8 mg/mL. The mean diameter of the LNP after sonication was determined by dynamic light scattering (Zetasizer Nano ZS, Malvern Instruments Inc., Westborough, MA). The LNP-DNA or LNP-siRNA encapsulated efficiency was quantified with a Quant-iT Pico-Green dsDNA assay kit (Thermo Fisher Scientific, P11496) or Quant-iT RiboGreen RNA Assay Kit (Thermo Fisher Scientific, R11490) according to the instructions. Before injection, the LNPs were briefly sonicated (3s for 3 times).

### Cytotoxicity assay

Peripheral blood mononuclear cells (PBMCs) were prepared from human buffy coats (Gulf Coast Regional Blood Center, Houston, TX, USA) using Ficoll-Hypaque (MilliporeSigma, Chicago, IL, USA). Tumor cells (1 × 10^4^) were incubated with E285K-mAb (10 µg/ml) for 30 min at 4°C, or cultured with LNP-pE285K-mAb (5 µg/ml) for 24 h at 37 °C. The treated tumor cells were then co-cultured with PBMCs with a ratio of 1: 50 in 96-well plates (Corning, NY, USA) at 37 °C for 72 h. Cytotoxicity was determined by measuring the amount of lactate dehydrogenase (LDH) in the supernatant using the Cytotoxicity Detection Kit PLUS (Roche, Indianapolis, IN, USA) according to the manufacturer’s instructions.

### Flow cytometry assessments

Apoptosis in MC38-p53^KO/E285K^, RPMI-8226, and BT-474 cells was analyzed using the APC Annexin V Apoptosis Detection Kit with 7-AAD (BioLegend, 640,930) according to the manufacturer’s instructions. The surface and intracellular expression of p53^E285K^ in MC38-p53^KO^ or MC38-p53^KO/E285K^ cells was evaluated by surface or intracellular staining using anti-p53-E285K-mAb and Alexa Fluor 647-conjugated goat anti-mouse IgG (H + L) secondary antibody (Invitrogen, A-21,235).

The tumor tissues were excised and minced into approximately 1 mm^3^ cubic pieces. They were then digested using a mouse Tumor Dissociation Kit (Miltenyi Biotec) and incubated on a rocker (Gentle MACS Octo 8, Miltenyi Biotec) at 37 °C for 25–40 min. The resulting digested cells were filtered through 70-µm cell strainers (BD Pharmingen) and washed twice with cold PBS containing 2% FBS. The total isolated cells were counted using a cell counter, and some cells were stained and detected by flow cytometry (FACS) to obtain the percentage of immune subsets after blocking Fc receptors and removing dead cells with a Zombie Aqua Fixable Viability Kit (BioLegend). Cell surface staining was performed by incubating with the following antibodies for 30 min at 4 °C, followed by intracellular staining. All data were obtained on a Cytek® NL-3000 FACS system (Cytek Biosciences) and analyzed using FlowJo V10 (BD Biosciences). The antibodies used in this study are as follows: anti-mouse CD16/32 (clone 93, BioLegend), Human TruStain FcX™ (BioLegend, 422,302), APC anti-human IgG Fc (clone M1310G05, BioLegend), Brilliant Violet 421™ anti-human CD45 (clone HI30, BioLegend), PE anti-human IFN-γ (clone W19227A, BioLegend), APC/Cy7 anti-mouse CD45 (clone 30-F11, BioLegend), Brilliant Violet 750™-conjugated anti-mouse CD45 (clone 30-F11, BioLegend), Percp/cy5.5 anti-mouse CD19 (clone 1D3/CD19, BioLegend), PE anti-mouse CD3 (clone 17A2, BioLegend), Pacific Blue™ anti-mouse CD4 (clone RM4-5, BioLegend), PerCP/Cyanine5.5 anti-mouse CD8a (clone 53 − 6.7, BioLegend), APC anti-mouse NK-1.1 (clone S17016D, BioLegend), APC-conjugated anti-mouse IFN-γ (clone XMG1.2, 505,810), FITC-conjugated anti-mouse TNF-α (clone MP6-XT22, Biolegend), PE-conjugated anti-mouse IL-2 (JES6-5H4, Biolegend), APC anti-mouse CD11c (clone N418, BioLegend), FITC anti-mouse/human CD11b (clone M1/70, BioLegend), PE anti-mouse CD103 (clone 2E7, BioLegend), PerCP anti-mouse F4/80 (clone BM8, BioLegend), Alexa Fluor® 647 anti-mouse FOXP3 (clone MF-14, BioLegend), FITC anti-mouse CD107a (clone 1D4B, BioLegend), PE anti-mouse/human CD44 (clone IM7, BioLegend), FITC anti-mouse CD62L (clone MEL-14, BioLegend), PE anti-mouse CD80 (clone 16-10A1, BioLegend), FITC anti-mouse CD86 (clone GL-1, BioLegend), Pacific Blue™ anti-mouse I-A/I-E (clone M5/114.15.2, BioLegend), PerCP/Cyanine5.5 anti-mouse H-2K^d^/H-2D^d^ (clone 34-1-2 S, BioLegend).

### Flow PLA

The Proximity Ligation Assay (PLA) was conducted following the Duolink PLA FACS protocol with modification using the Duolink flowPLA Detection Kit–Voilet (Sigma-Aldrich, DUO94005). MC38-p53^KO/E285K^ cells were washed, fixed, and permeabilized. Following three PBS washes, samples were blocked with Duolink Blocking Solution (Sigma-Aldrich, DUO82007) for 1 h at 37 °C. Primary antibodies, including the purified E285K-mAb and TRIM21-mAb (Abcam, ab207728), were added at 5 µg/mL in antibody dilution solution at 37 °C for 1 h. After washing twice with PBS, samples were incubated with the Duolink In Situ PLA Probe Anti-Mouse MINUS (Sigma-Aldrich, DUO92001) and Duolink In Situ PLA Probe Anti-Rabbit PLUS (Sigma-Aldrich, DUO92005) in PLA antibody diluent (MilliporeSigma, DUO82008) for 1 h at 37 °C. Following another PBS wash, ligase (1:40 in Duolink Ligation buffer) was added to the cells and incubated for 30 min at 37 °C to facilitate ligation. After washing twice with Duolink Wash Buffer A, the cells were incubated with DNA polymerase (1:80 in amplification buffer) overnight at 37 °C. Following two washes with Duolink Wash Buffer, the cells were incubated for 30 min at 37 °C with 1× flowPLA Detection Solution. After two additional washes with Duolink Wash Buffer B, the cells were subjected to measurement using the Cytek® Northern Lights cytometer (Cytek Biosciences).

### Animal experiments

A syngeneic mouse colon cancer model was established by subcutaneous inoculation of MC38-p53^KO/E285K^ cells (3 × 10^5^ cells/mouse). The LNP-pE285K-mAb (IgG1 or IgA) or control plasmid (40 µg/mouse) was administered intratumorally at specified time points. Meanwhile, combination therapy with 250 µg/mouse αPD-1 (BioXcell, BE0146) or 500 µg/mouse αCD4 (BE0119), αCD8 (BP0117), αNK1.1(BE0036), and αCD19 (BE0150) was intraperitoneally administered after intratumor injection. For the lung metastasis model, 1 × 10^6^ MC38-p53^KO/E285K^ cells were injected into the tail vein on day 0. The mice were given an intravenous injection of the same amount of LNP-pE285K-mAb (40 µg/mouse) on day 7 and repeated treatment on day 12 post tumor inoculation. On day 22 post tumor inoculation, mice were sacrificed, and the lungs were surgically excised. Metastatic nodules in the lung tissue were quantified by observing histological sections of the entire transverse plane of lung tumors in each mouse and calculating the number of tumor metastatic foci. To establish intestinal tumors, the mice were injected intraperitoneally with 2 × 10^6^ MC38-p53^KO/E285K^ cells suspended in 100 µl of PBS. The mice were then intraperitoneally treated with LNP-pE285K-mAb on days 7 and 14. Tumor growth was monitored, and animal survival was evaluated. To calculate the tumor inhibition rate, the following formula was used: (C-T)/C×100%; T is the tumor weight from each animal, and C is the weight of the largest tumor from the control group.

NSG mice were subcutaneously inoculated with RPMI-8226 cells or BT-474 (5 × 10^6^ cells/mouse) resuspended in a mixture of serum-free medium and Matrigel (Corning, 354,230) at a 1:1 volume ratio into the right flank. On day 10 post-tumor inoculation, 1 × 10^7^ PBMCs were administered intravenously to each mouse. Fourteen days post tumor inoculation, 5 mice were randomly grouped and treated with either an intratumor injection of LNP-pE285K-mAb or LNP-Ctrl (40 µg/mouse) on days 14, 19 and 24. Similarly, Hu-NSG-CD34 mice were subcutaneously inoculated with BT-474 (5 × 10^6^ cells/mouse) and intratumorally treated on days 14 and 21 post tumor inoculation. Humane endpoints were defined as tumor length reaching 1.5 cm, tumor burden equal to or greater than 10% of the normal body weight, or severe tumor necrosis. The tumor burden and mouse weight were measured, and tumor volume was calculated using the following equation: V = (length × width^2^)/2. All procedures were performed with the approval of the Institutional Animal Care and Use Committee of the Baylor College of Medicine.

### scRNA-seq sequencing

Subcutaneous tumors were harvested 14 days after a single-dose treatment, excised, and minced in RPMI-1640 medium supplemented with 10% FBS. The dissected tumor samples were dissociated into single-cell suspension using a mouse Tumor Dissociation Kit (Miltenyi Biotech, 130-096-730) with a gentleMACS Octo Dissociator with Heaters (Miltenyi Biotech). Cells were collected by using a 40 μm cell strainer (Corning, 431,750), centrifuged at 300 g for 10 min, and lysis red cells using ACK buffer, and resuspended in RPMI1640with 5% FBS. Single-cell suspensions were refined by excluding dead cells using Zombie Green viability dye, and Fc receptors were blocked before the addition of APC-Cy7-conjugated anti-mouse CD45 antibody. Subsequently, CD45^+^ cells were sorted from each tumor sample using a FACSAria II instrument. Equivalent numbers of sorted CD45^+^ cells from five mice per group were combined, concentrated by centrifugation, and resuspended at a density of 1 × 10^3^ cells/µl in RPMI1640 medium containing 10% FBS. Approximately 10,000 cells were allocated for the 10 × Genomics 5′ v2 single-cell assay for both experimental groups. TCR/BCR libraries were prepared using the Chromium™ single cell V(D)J enrichment kit (10× Genomics). Libraries were prepared according to the manufacturer’s protocol at the Single Cell Genomics Core at Baylor College of Medicine (BCM). BCM Genomic and RNA Profiling (GARP) Core sequenced the libraries on NovaSeq 6000.

### Processing of scRNA seq data

Raw sequence reads in the FASTQ formats were aligned to the mouse reference genome using CellRanger Count v7.1.0 pipeline (https://cloud.10xgenomics.com) with the default settings for alignment, barcode assignment, and UMI counting of the raw sequencing data with the genome reference Mouse (mm10) 2020-A. BCR and TCR raw gene expression matrices were generated using the CellRanger count pipeline with default parameters and mouse GRCm38/mm10 as the reference genome.

Doublet cells were eliminated using the R package scDblFinder(1.14.0) [29], and additional processing was performed using the R package Seurat (v4.4.0) [30]. Filtered cells had > 10% mitochondrial, nFeature RNA > 7500, or < 200 identified genes. After log-normalization, the highly variable genes were determined using the “vst” selection method in Seurat and the FindVariableFeatures tool. The ScaleData function in Seurat was used to scale the data. The RunHarmony function from the R package harmony (1.01) [31] combined multiple data after PCA analysis. Neighbors and clusters were defined using Seurat’s FindNeighbors and FindClusters function and a resolution of 0.5. RunUMAP was used to calculate the UMAP dimensionality reduction with default parameters utilizing the top 30 principal components (PCs) and harmony reduction. For the analysis focusing on T cells, B cells, NK cells, and NK-like cells, which are subsets of the annotation cell types, data were re-run from the NormalizeData function following the Seurat pipeline. To assist with annotating the T cell subcluster, we utilize the R packages STACAS (2.1.3) [32] and ProjecTILs (3.2.0) [33].

### Differentially expressed genes (DEGs) and enrichment analysis

DEGs between clusters were obtained with a Wilcoxon followed by Bonferroni correction using the FindAllMarkers function in Seurat. For GO and KEGG enrichment analysis, Clusterprofiler (4.8.3) [34] was used to examine DEGs in cell clusters. The AddModuleScore function was used to evaluate the gene set score of T cell transcription factors, memory markers, cytotoxic markers, and checkpoint markers. To plot specific genes or gene set scores of interest, the FeaturePlot function in Seurat and the plot_density function in Nebulosa (1.10.0) [35] were used to show the genes or gene set score distribution on cells. UMAP and heatmap plot modification was done using scRNAtoolVis (0.0.7).

### Processing of scVDJ seq data

scVDJ and scRNA seq analysis were performed using the Seurat and scRepertoire (1.11.0) [36] packages. In brief, VDJ sequences were extracted using the combine TCR or combine BCR function in scRepertoire. We used the Python module CoNGA to calculate and show the TCR motifs within TRAV and TRAB from T cells subclusters.

### Pseudotime analysis

Pseudotime analysis was performed via two different methods. R package FitDevo (1.2.1) [37] was used to analyze the developmental trajectories of T, B, NK, and NK-like cells and to infer the CD8^+^ and CD4^+^ T cell starting cell populations through global cell differentiation. R package Slingshot (2.8.0) [38] was used to infer the differentiation paths of CD8^+^ T cells and CD4^+^ T cells. Combining the UMAP dimensionality reduction results in Seurat and the starting trajectory of FitDevo analysis, we used the slingPseudotime function to predict the cell differentiation path and the potential developmental pathways in each cell group.

### Receptor–ligand interaction analysis

The CellChatR toolkit (2.2) [39] was used to explore cellular interactions different between control and treatment groups. Communication probabilities were calculated based on the CellChatDB database of literature and PPI-supported ligand–receptor interactions in mouse datasets. First, we filtered the incoming and outgoing parameters for each sample using the selectK function before aggregating the data from the two cell groups using the mergeCellChat function. The Cellchat protocol “Comparison analysis of multiple datasets using CellChat” was used to analyze the merged data items.

### Statistical analyses

Unless otherwise stated, data were expressed as the mean ± standard deviation (SD). Two groups were compared using a two-tailed Student’s *t*-test. GraphPad Prism 8.0 (GraphPad Software, San Diego, CA, USA) was used for statistical analysis. Values of *p* < 0.05 were considered statistically significant (*p < 0.05, **p < 0.01, ***p < 0.001, ****p < 0.0001).

## Results

### E285K-mAb and LNP-pE285K-mAb are cytotoxic to tumor cells with p53^E285K^

**Fig. 1:**
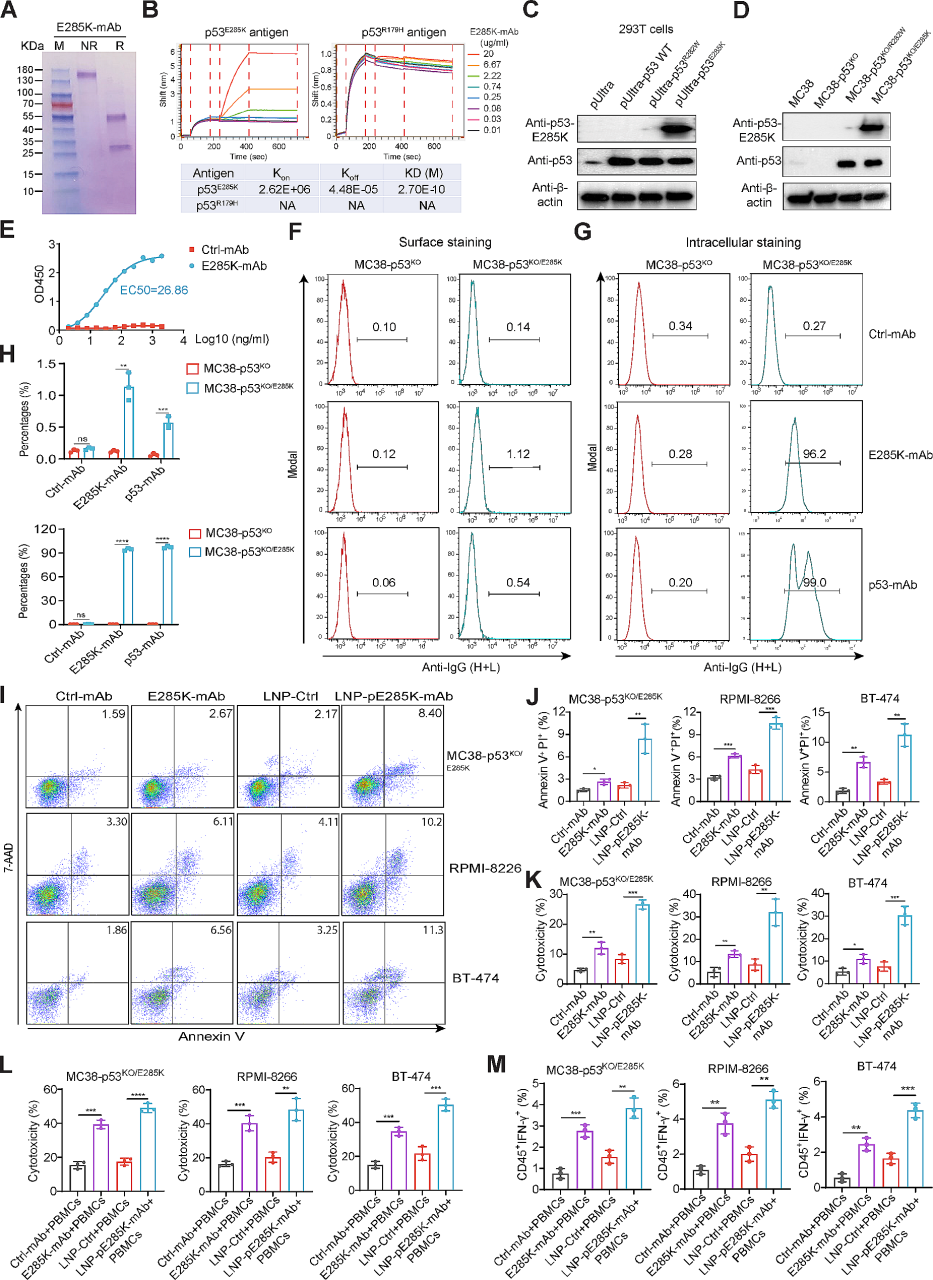
The cytotoxicity of E285K-mAb and LNP-pE285K-mAb to tumor cells with p53^E285K^. **A** Purified E285K-mAb was analyzed on an SDS-PAGE gel and visualized with Coomassie staining. Lane M: Protein ladder; Lane NR: Non-reducing; Lane R: Reducing. **B** BLI kinetics of E285K-mAb binding to the antigen. **C** Western blot analysis of WT p53, p53^E285K^, and p53^R282W^ in 293T cells 48 h post transfection. **D** Western blot of p53^E285K^ in MC38-p53^KO/E285K^ cells (p53-null MC38 cells with exogenous p53^E285K^). **E** ELISA of E285K-mAb with the antigen. The “Ctrl-mAb” refers to HEL-mAb, which contains a human Fc and a Fab targeting the hen egg lysozyme (HEL). **F-H** FACS of MC38-p53^KO/E285K^ cells using E285K-mAb. Cells were treated with the BD Cytofix™ Fixation Buffer in (**F**) and with the Cytofix/Cytoperm™ Fixation/Permeabilization Kit in (**G**). Bar graphs were shown (**H**). **I** and **J** FACS to analyze cellular apoptosis of MC38-p53^KO/E285K^, RPIM-8226, and BT-474 cells. Both RPIM-8226 and BT-474 cell lines harbor endogenous p53^E285K^ mutation. Cells were treated with Ctrl-mAb, E285K-mAb, LNP-Ctrl, or LNP-pE285K-mAb for 72 h. **K** and **L** Cytotoxicity of cells treated with mAbs and PBMCs. Cells were treated with E285K-mAb (10 µg/ml) for 30 min at 4 °C or LNP-pE285K-mAb (5 µg/ml) for 24 h at 37 °C before co-cultured with PBMCs at a 50:1 E: T ratio in 96-well plates for 72 h. Cytotoxicity was measured by an LDH release assay. **M**, IFN-γ expression in PBMCs co-cultured with cancer cells and mAbs. Data were representative of three experiments. Presented as means ± SD. Statistical significance: *p < 0.05, **p < 0.01, and ****p < 0.0001; ns, not significant

We used the mouse hybridoma method to screen mAbs against p53^E285K^ and obtained six clones (619, 882, 1002, 1250, 1331, 1408). Immunoprecipitation, BLI, and western blotting revealed that clone 1331 exhibited the best affinity and specificity for the mutant epitope (Supplementary Fig. S1A-C). We constructed plasmids encoding the HL and LC of the mouse mAb with a human IgG1 Fc fragment to express the mAb in 293Expi cells. The mAb was purified (Fig. 1A), and its binding affinity was determined using BLI with a dissociation constant (Kd) of 27 pM (Fig. 1B). The mAb displayed strong recognition of p53^E285K^ but not of the WT p53 or p53^R282W^ protein (Fig. 1C-E). FACS without cell permeabilization indicated that the mAb recognized a small number of MC38-p53^KO^ cells expressing exogenous human p53^E285K^ (Fig. 1F and H). Intracellular staining after permeabilization revealed specific binding of the mAb to mutant p53^E285K^ protein without cross-reaction with p53^R282W^ (Fig. 1G and H).

Adding the mAbs in purified recombinant protein (E285K-mAb) or in encoding plasmids packaged in LNPs (LNP-pE285K-mAb) into cultured MC38-p53^KO/E285K^, RPMI-8226, and BT474 cells (with endogenous p53^E285K^) induced apoptosis (Fig. 1I and J). E285K-mAb moderately reduced cell proliferation, whereas LNP-pE285K-mAb exhibited robust cytotoxicity, as measured by LDH release (Fig. 1K). When tumor cells were co-cultured with PBMCs, both E285K-mAb and LNP-E285K-mAb showed significantly increased cytotoxicity (Fig. 1L). Furthermore, the levels of IFN-γ, a mediator of cytotoxicity secreted by immune cells, were increased in PBMCs co-cultured with tumor cells and treated with E285K-mAb or LNP-pE285K-mAb (Fig. 1M). These results indicate that E285K-mAb and LNP-pE285K-mAb are cytotoxic to tumor cells with p53^E285K^, which is augmented by immune cells.

### The LNP-pE285K-mAb treatment exhibits potent inhibition of tumor growth and promotes T cell infiltration in the TME

**Fig. 2:**
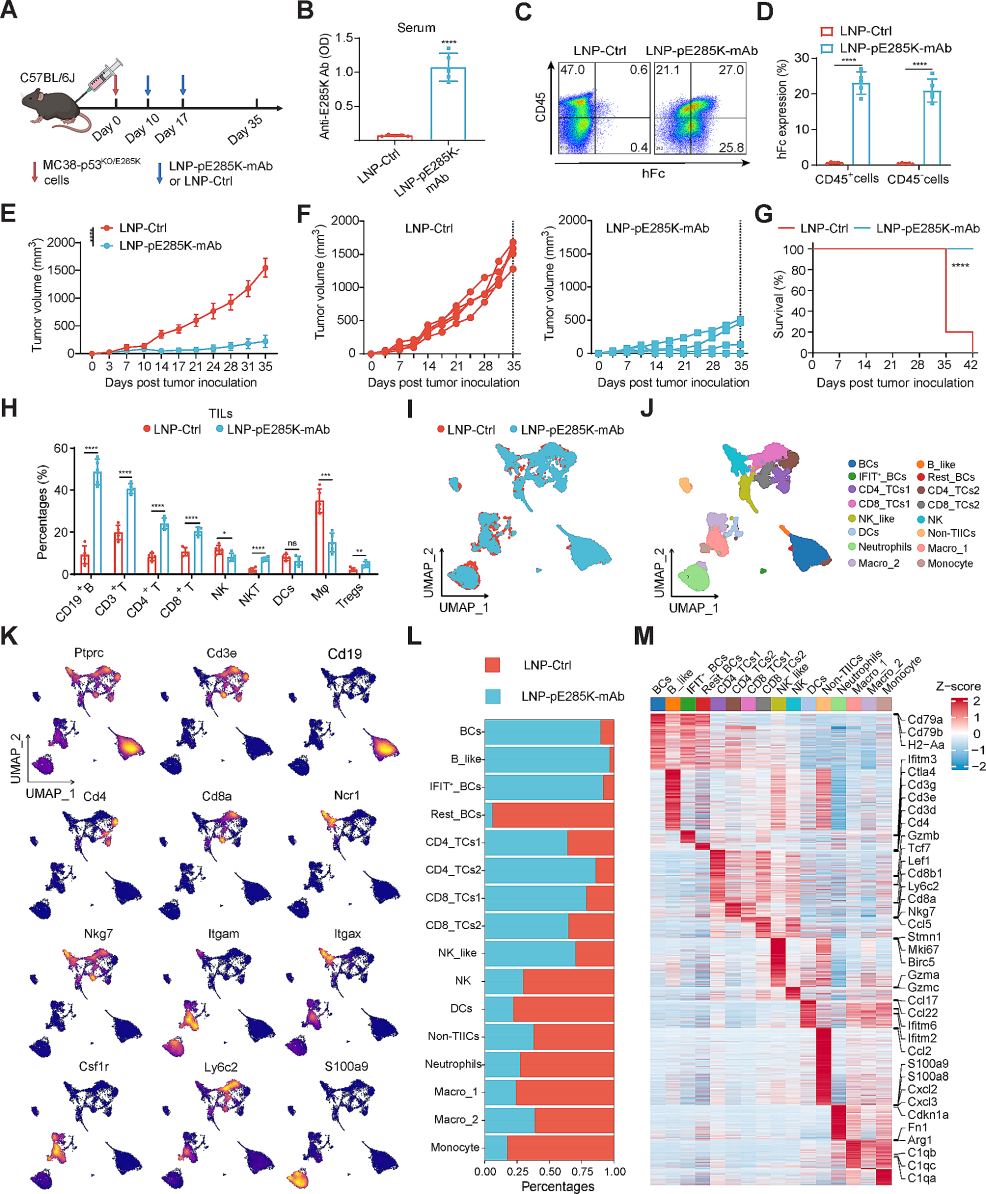
Therapeutic efficacy of LNP-pE285K-mAb to treat MC38-p53^KO/E285K^tumors. **A** Schematic representation of the subcutaneous tumor model. Established tumors from MC38-p53^KO/E285K^ cells were treated by intratumoral injection of 40 µg DNA plasmids encoding pE285K-mAb (20 µg each for light-chain and heavy-chain) per tumor 10 days after tumor cell inoculation (*n* = 5 mice). **B** Serum levels of E285K-mAb detected using ELISA. **C** and **D** FACS analysis of E285K-mAb expression and binding in tumors using anti-hFc mAb. **E** Tumor volumes in each group were measured at different times after inoculation. The initial tumor size was approximately 50–100 mm^3^, and treatment with LNP-pE285K-mAb began 10 days after tumor inoculation. **F** The volumes of each tumor. **G** Survival rate of the two mouse groups (*n* = 10 mice). **H** Proportions of CD19^+^B, CD3^+^T, CD4^+^T, CD8^+^T, NK, NKT, DCs, Mφ, and Tregs in TILs from the two groups. **I** and **J** scRNA-seq of CD45^+^ immune cells isolated from two groups of tumors, visualized through unified manifold approximation and projection (UMAP). **K** Identification expression of representative marker genes, such as Ptprc (Cd45) for pan-leukocytes and Cd19 for B cells. **L** Immune cell subtype changes in tumors treated with LNP-pE285K-mAb. **M** Comparative analysis of DEGs in immune cells from the two groups. Data were presented as means ± SD. Statistical significance was set at *p < 0.05, **p < 0.01, and ****p < 0.0001; ns, not significant

Next, we evaluated the anti-tumor activity of LNP-pE285K-mAb in vivo. We treated mice carrying an established tumor from MC38-p53^KO/E285K^ with two doses of LNP-pE285K-mAb intratumorally (Fig. 2A). The LNP-pE285K-mAb-treated group showed a significant increase in anti-E285K-mAb serum levels on day 5 after the first dose (Fig. 2B). To assess whether the treatment targeted the mutant antigen presented on tumor cells in the TME, we performed FACS analysis on cells isolated from tumors. The LNP-pE285K-mAb-treated group displayed increased staining by the anti-Fc antibody for CD45^−^ and CD45^+^ cells (Fig. 2C and D). These results indicated the mAb was expressed or displayed by both tumor cells and immune cells. The MC38 tumors generally consist of > 80% tumor cells. The mAbs displayed on immune cells may be secreted by tumor cells and bound to immune cells with Fc receptors or expressed by immune cells that receive the LNPs. The intratumoral injection of LNP-pE285K-mAb effectively inhibited tumor growth (Fig. 2E). By day 42 post-tumor inoculation, the treated mice exhibited a 100% survival rate with 40% complete response (CR), compared to zero in the control group (Fig. 2F and G). The treatment caused no apparent inflammatory reactions or lesions in vital organs such as the heart, liver, spleen, lung, and kidney (Supplementary Fig. S2A). The cytokine levels in mice temporarily increased on the second day post-LNP injection and returned to normal after one week, indicating a transient cytokine elevation (Supplementary Fig. S2B). The control (LNP-HEL-mAb) moderately inhibited tumor growth but caused no CR (Supplementary Fig. S2C and D). LNPs may serve as adjuvants and plasmids as STING agonists in the control. These findings affirm the therapeutic efficacy and safety of this LNP-mediated antibody therapy.

Tumor-infiltrating immune cells (TIICs), including B, T, and NK cells, play a pivotal role in tumor-controlling. We treated another cohort of animals with xenograft tumors with a single dose of LNP-pE285K-mAb, which caused a significant increase of immune cell subsets, such as B, T, and NKT cells, within the tumors compared to the control group, as measured by FACS (Fig. 2H). We next performed single-cell sequencing (scRNA-seq) of CD45^+^ immune cells extracted from the tumors and defined 16 immune cell subsets: BCs (B cells), B_like (B-like cells), IFIT^+^_BCs, Rest_BCs, CD4_TCs1 (CD4^+^T cell cluster 1), CD4_TCs1 (CD4^+^T cell cluster 2), CD8_TCs1 (CD8^+^T cell cluster 1), CD8_TCs1 (CD8^+^T cell cluster 2), NK_like (NK-like cells), NK, DCs, non-TIICs, Neutrophils, Macro_1 (Macrophage subset 1), Macro_2 (Macrophage cluster 2) and monocytes (Fig. 2I-M, Supplementary Fig. S3A and B, and Supplementary Table 1), with percentages largely consistent with those obtained using FACS. UMAP analysis revealed a notable increase in B, B-like, IFIT^+^ B, CD4^+^ T, CD8^+^ T, and NK-like cells and a decrease of resting B cells, NK cells, DCs, non-TIICs, neutrophils, macrophages, and monocytes (Fig. 2L). KEGG and GO enrichment analyses highlighted significant antigen presentation by B cells and DCs, immune receptor reaction by NK-like cells, and activation of CD8^+^ T cells (Supplementary Fig. S3C and D). These results suggest that the LNP-pE285K-mAb enhances T cell infiltration and shifts them towards the anti-tumor subpopulations.

### LNP-pE285K-mAb suppresses tumor metastasis in syngeneic immunocompetent mice and xenograft tumorigenesis in humanized mice

**Fig. 3:**
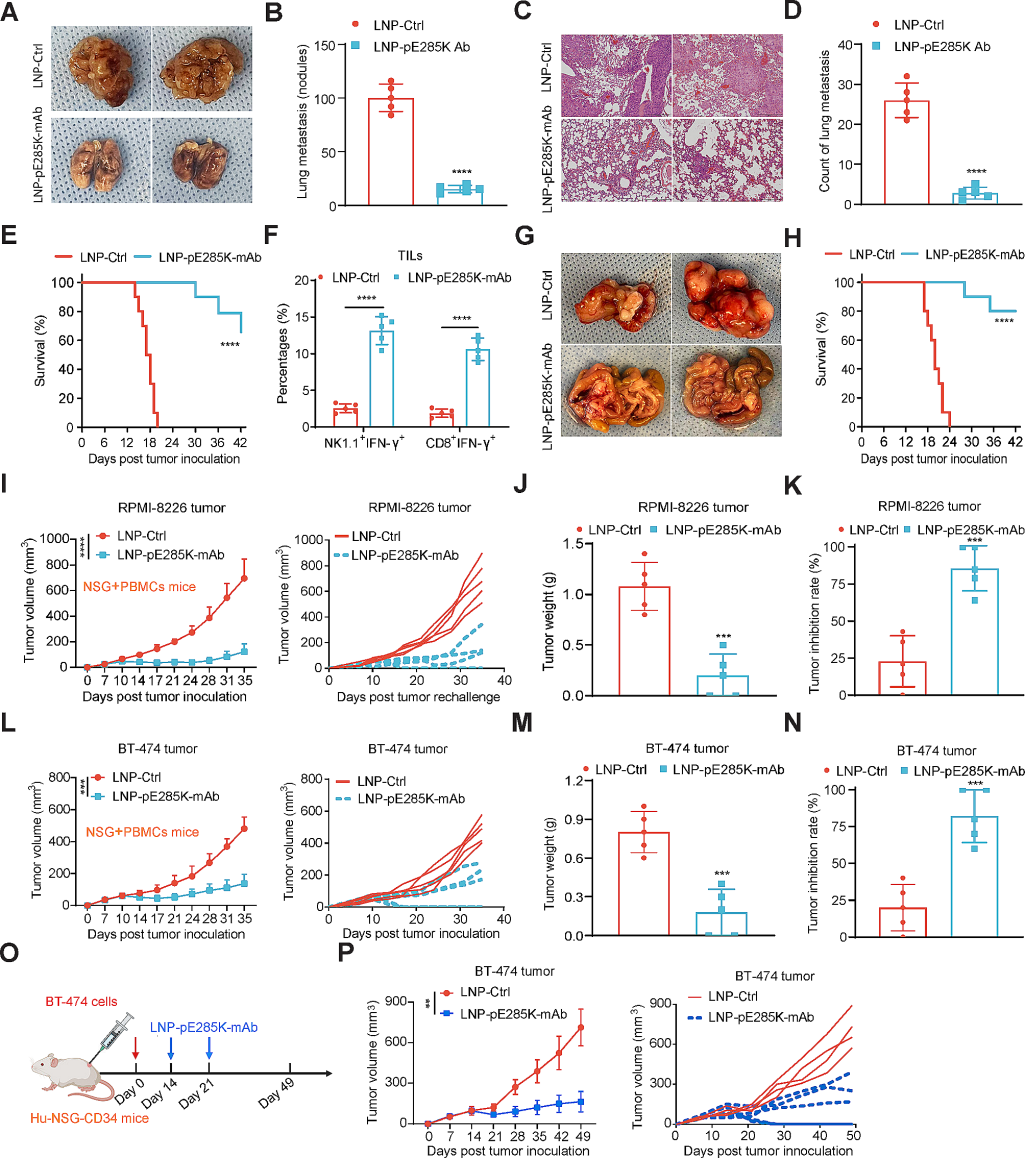
Anti-tumor response induced by LNP-pE285K-mAb in lung metastasis. **A** Representative images of metastatic nodules on the lung surface in different treatment groups. **B** The number of visible nodules present on the lung surface (*n* = 5 mice). **C** Histological examination of lung tissues. **D** Enumeration of microscopic lung metastases as identified in (**C**). **E** Survival analysis in mice with lung metastatic tumors (*n* = 10 mice). **F** The percentages of IFN-γ expression in NK or CD8^+^ T cells within TILs from lung metastatic tumors. **G** Representative images of rectal MC38-p53^KO/E85K^ tumors. **H** Survival analysis of mice with rectal MC38-p53^KO/E285K^ tumors (*n* = 10 mice). **I** Tumor development from RPMI-8226 cells. NSG mice were inoculated RPMI-8226 cells on day 0, treated by 1 × 10^7^ PBMCs/mouse intravenously on day 10 and LNP-pE285K-mAb on day 14 (*n* = 5 mice). **J** and **K** Tumor weight and inhibition on day 45 post inoculation of RPMI-8226 cells. **L** BT-474 tumor development (*n* = 5 mice). **M** and **N** Tumor weight and inhibition in BT-474 models. **O** Schematic representation of LNP-pE285K-mAb treatment in the Hu-NSG-CD34 tumor model. Hu-NSG-CD34 mice with BT-474 tumors were treated by intratumoral injection of LNP-pE285K-mAb (40 µg DNA plasmids) per tumor 14 and 21 days after tumor cell inoculation. **P** BT-474 tumor growth (LNP-Ctrl group, *n* = 4 mice; LNP-pE285K-mAb group, *n* = 5 mice). Data were represented as means ± SD. Statistical significance was set at ***p < 0.001, ****p < 0.0001

Next, we assess the therapeutic effect of LNP-pE285K-mAb on metastasis with MC38-p53^KO/E285K^ cells injected into the mouse tail vein. Mice were treated on days 7 and 12, and lung metastases were analyzed on day 22. The two-dose LNP-pE285K-mAb treatment significantly reduced the number of visible metastatic nodules (Fig. 3A and B). Histological examination further confirmed the protective effect of the treatment (Fig. 3C and D). All the mice in the control group succumbed by day 22, when all treated mice were alive. By day 42, the survival rate of the treated group remained at 70% (Fig. 3E). We found a significant augmentation in IFN-g^+^ NK cells and CD8^+^ T cells from lung metastases (Fig. 3F). We then injected tumor cells intraperitoneally (i.p.) and treated the animals with two i.p. doses of LNP-pE285K-mAb. There was a significant inhibition of intestinal tumor growth in situ and an improvement in the survival rate of the treated group (Fig. 3G and H). To validate its potential clinical utility, we treated immunodeficient NSG mice xenografted with RPMI-8226 or BT-474 cells, PBMCs, and the LNP-pE285K-mAb. We found that this treatment significantly inhibited the growth of RPMI-8226 and BT-474 tumors (Fig. 3I-N). We then inoculated BT-474 cells into Hu-NSG-CD34 mice, which are NSG mice engrafted with human cord blood-derived CD34^+^ hematopoietic stem cells. LNP-pE285K-mAb treatment suppressed the BT-474 tumor growth in Hu-NSG-CD34 mice (Fig. 3O and P). These results indicate the LNP-pE285K-mAb inhibits the development of lung and intestinal metastasis from mouse tumor cells and xenograft tumorigenesis from human cancer cells.

### The therapeutic effect of LNP-pE285K-mAb in TME primarily depends on CD8^+^ T cell response regulated by B cell subsets

**Fig. 4:**
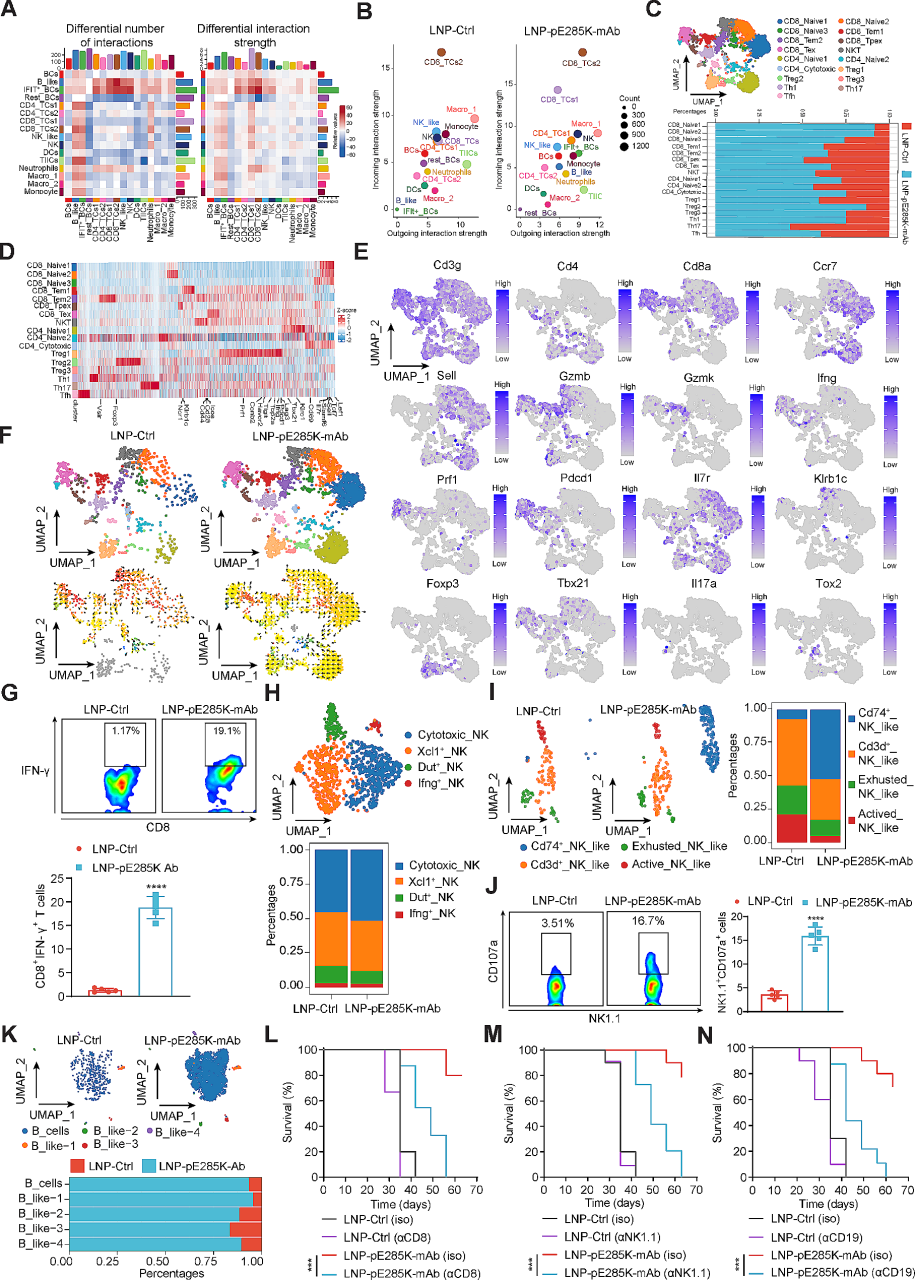
B cells and NK-like cells interacting with CD8^+^ T cells mediated by LNP-pE285K-mAb in TME. **A** Heat map depicting the differential number (left) and strength (right) of interactions in the cell-cell communication network between the LNP-pE285K-mAb-treated and LNP-Ctrl-treated groups. Red indicates up-regulated signaling, and blue indicates downregulated signaling. **B** Scatter plots comparing the outgoing and incoming interaction strengths in the 2D space between groups. **C** UMAP visualization of T cell-associated populations pooled across samples and conditions, clustered using the Louvain algorithm into 17 distinct clusters. Bar graphs showed the percentages of various T cell subtypes in the tumors from each group. **D** Heatmap presenting DEGs in T cells between the groups. **E** Expression profiles of signature genes (from **C**) and genes crucial for T cell function. **F** Developmental trajectory analysis indicating the dynamic shift in cell states, with arrows predicting the direction of cell state transition. **G** Proportion of IFN-γ expression in CD8^+^ T cells from TILs. Shown were representative FACS results from one of three experiments (*n* = 5 mice). **H** UMAP projections illustrating NK cell subtypes and their heterogeneity in tumors from each group. **I** UMAP plot of several NK-like cell subtypes. **J** Percentages of CD107a expression in NK1.1^+^ cells from TILs evaluated by FACS (*n* = 5 mice). **K** UMAP projections of B cell subtypes within tumors across groups. **L-N**, The impact of blocking CD8^+^ T, NK, and B cells on animal survival. Each animal (*n* = 10 per group) was intraperitoneally injected with 0.5 mg anti-mouse CD8α, CD19, or NK1.1 mAb 2 days before the first dose of LNP-pE285K-mAb (second and third on days 5 and 12). Data were represented as means ± SD. Statistical significance was set at ***p < 0.001

Based on scRNA-seq data, a notable increase in the number and strength of interactions was observed between B-like, IFIT^+^ B, and various T, NK, and NK-like cell groups (CD4_TCs1, CD4_TCs2, CD8_TCs1, CD8_TCs2, NK, and NK_like, respectively) in the treated group. Notably, the outgoing and incoming signaling of IFIT^+^ B, B-like, and two CD8^+^ T cell clusters was consistently strengthened, aligning with the role of B cell-mediated CD8^+^ T cell immune responses in anti-tumor immunity. Myeloid cell populations, including macrophages, monocytes, non-TIICs, and resting B cells, exhibited significantly decreased outgoing and incoming signaling strength, while NK and CD4_TCs1 showed a slight increase in interaction strength (Fig. 4A and B). The LNP-pE285K-mAb treatment led to a significant increase in the ratios of CD8_Naive, CD8_Tem (Memory-like CD8^+^T), CD8_Tex (Exhausted CD8^+^ T), CD4_Cytotoxic, and Th1 (T helper 1), as well as a decrease in Th17 (T helper 17) cells (Fig. 4C). Enrichment analysis confirmed the enrichment of T cell activation-related signaling pathways (Fig. 4D and E, and Supplementary Fig. S4A and B). There was a dynamic shift towards CD8_Tem1, CD8_Tem2, and CD8_Tpex (progenitor exhausted CD8^+^ T) cells in the treated group, suggesting the induction of memory and stem-like CD8^+^ T cells that are capable of expansion, regeneration, and tumor-killing (Fig. 4F). CD8_Tpex is a T cell subset that exhibits robust effector functions and potent cytolytic capabilities for tumor cell elimination. Intracellular staining and FACS demonstrated an increased percentage of CD8^+^ T cells secreting cytotoxic cytokines IFN-γ upon treatment (Fig. 4G), indicating enhanced induction of tumor antigen-specific CD8^+^ T cell responses.

We also observed a strong communication between B cells and NK and NK-like cells in response to the LNP-pE285K-mAb treatment. Four clusters of NK cells were identified through marker gene expression and enrichment analysis: Cytotoxic_NK, Xcl1^+^_ NK, Dut^+^_NK, and Ifng^+^_NK cells (Fig. 4H and Supplementary Fig. S5A, C, and D). These were enriched in the treated group, with cytotoxic_NK being the predominant cluster (Fig. 4H). Developmental inference revealed a dynamic shift towards cytotoxic_NK cells in the treated group (Supplementary Fig. S5E). XCL1, a chemokine to recruit XCR1^+^ conventional DC type 1 (cDC1) cells to tumors, was significantly upregulated in NK and double-negative T cells in the treated group (Fig. 4H and Supplementary Fig. S5A). The treatment also caused a significant increase in Cd74^+^_NK_like cells and a decrease in Cd3^+^_NK_like, exhausted_NK_like and active_NK_like cells (Fig. 4I and Supplementary Fig. S5B-D). CD74 is an integral membrane protein expressed by most B-cells and functions as an MHC-II chaperone. Cd74^+^_NK_like cells not only exhibit NK cell-mediated toxicity but also present antigens in a manner similar to B cells. Developmental inference showed a dynamic differentiation shift to Cd3^+^_NK_like and exhausted_NK_like cells in both groups (Supplementary Fig. S5F). Cytotoxic NK cells were detected in TIICs, with increased percentages of CD107a^+^ NK cells in the treated group, as determined by FACS (Fig. 4J). The treatment promoted an increase in all B cell subsets and B-like cell clusters (B-like_1,2,3,4; Fig. 4K, Supplementary Fig. S6A-E), with a dynamic shift towards B and B-like_1 in cell state (Supplementary Fig. S6D-F). Cell depletion using blocking antibodies demonstrated that CD8^+^ T, NK, and B cells are pivotal to the anti-tumor immunity induced by LNP-pE285K-mAb (Fig. 4L-N). Th1 response is critical to the activation of CD8^+^ cytotoxic T cells to target and destroy tumors, yet the depletion of CD4^+^ T cells did not affect the therapeutic benefit of LNP-pE285K-mAb (Supplementary Fig. S7A-H). Thus, multiple effector cells contribute to the efficacy of the LNP-pE285K-mAb treatment.

### LNP-pE285K-mAb exerts a long-lasting anti-tumor effect by CD8^+^ T cells

**Fig. 5:**
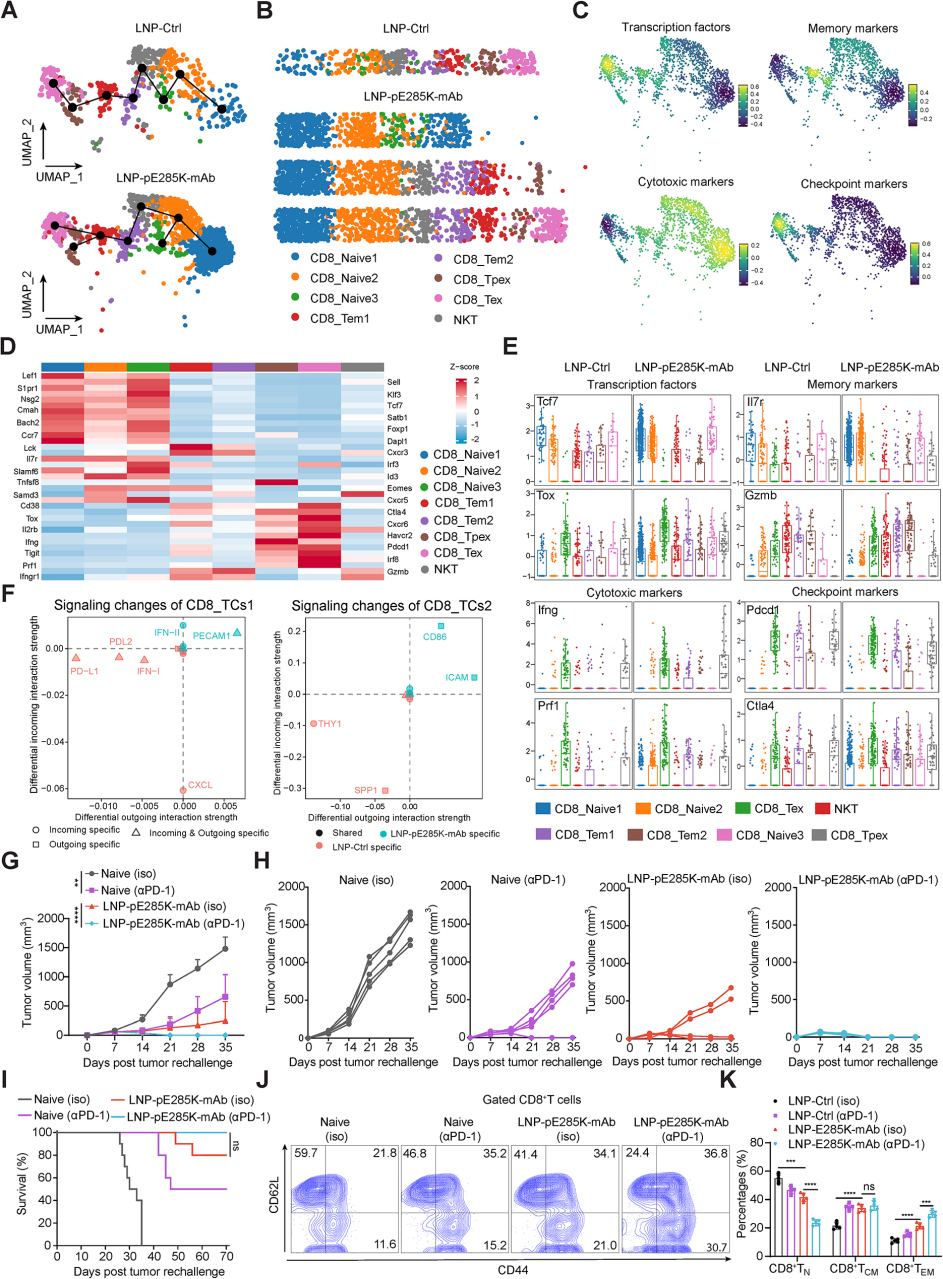
scRNA-seq unveils unique CD8^+^ T cell subpopulation induced by LNP-pE285K-mAb. **A** UMAP representation delineating the developmental trajectory of Tex, Tpex, Tem1 and Tem2 cells within CD8^+^ T cell populations. **B** Pseudotime analysis of the states indicated in (**A**). **C** Quantification and expression intensity of marker genes from (**A**) superimposed on the UMAP plot. **D** Heatmap displaying the top DEGs in CD8^+^ T cell subpopulations and NKT cells. **E** Boxplots showing the expression of genes like transcription factor, memory, effector, and checkpoint markers. Data were analyzed using the Kruskal-Wallis test. **F** Identification of specific signaling alterations in two CD8^+^ T cell subtypes between the two groups. **G** The contribution of PD-1 blockade to the long-term therapeutic efficacy of LNP-pE285K-mAb. comparison in mice treated with LNP-pE285K-mAb, αPD-1, or their combination, followed by challenge with MC38-p53^E285K^ tumor cells (*n* = 5 mice per group). **H** Tumor volumes of individual mice from (**G**). **I** Animal survival (*n* = 10 mice per group). **J** Representative images of FACS of CD44 and CD62L expression in CD8^+^ T cells from different groups. **K** Statistical analysis of the percentages of naive (CD44^−^CD62L^high^), central memory (CD44^+^CD62L^high^), and effector memory (CD44^+^CD62L^low^) CD8^+^ T cells in (**J**). Data were from one representative of three experiments, presented as mean ± SD. Statistical significance was set at **p < 0.01, ***p < 0.001 and ****p < 0.0001; ns, not significant

The developmental inference of CD8^+^ T cells towards CD8_Tem1, CD8_Tem2, and CD8_Tpex is crucial for the therapeutic effect of LNP-pE285K-mAb. To further understand the differentiation trajectories of CD8^+^ T cell subtypes, pseudotime analyses were conducted. In the control group, only one differentiation route was observed, starting with CD8_Naive1 cells and bifurcating into NKT, CD8_Tem1, CD8_Tem2, CD8_Tpex, and CD8_Tex cells. Three differentiation routes emerged in the treated group (Fig. 5A and B). The diversity and multifunctionality of CD8^+^ T cells are helpful for tumor control. Principal components analysis (PCA) of function markers for transcription factors, T cell memory, cytotoxicity, and checkpoints by scRNAseq was visualized as UMAP plots in Fig. 5C. The mRNA levels of DEGs in CD8^+^ T cell clusters were shown in Fig. 5D and E. To identify the signaling pathways contributing to the dramatic signaling changes in immune regulation, we calculated the differential outgoing and incoming interaction strength of each signaling pathway between CD8_TCs1 and CD8_TCs2. In the LNP-pE285K-mAb-treated group, IFN-II and PECAM1 incoming signaling emerged as the most predominantly increased signals in CD8_TCs1, whereas CD86 and ICAM outgoing signaling dominated in CD8_TCs2 compared to the control group (Fig. 5F). These largest differential outgoing and incoming interaction strengths indicated an increased induction of activated CD8^+^ T cells and a decreased induction of exhausted CD8^+^ T cells in LNP-pE285K-mAb treatment, in agreement with the previous findings (Fig. 4A and B). In addition to PD-L1 and PD-L2 signaling, we observed increased signaling changes in both outgoing and incoming signaling in CD8_TCs1. For the control group, increased THY1 and SPP1 levels were observed in CD8_TCs2 (Fig. 5F). PD-L1, PD-L2, THY1, and SPP1 from tumor or immune-inhibited cells play a central role in inhibiting the function of CD8_Tpex and CD8_Tex cells during anti-tumor immunity. This inhibition impedes the expansion of CD8^+^ T cells and the induction of memory CD8^+^ T cells [40]. Indeed, our analysis showed that the contribution of each ligand–receptor pair to the PD-1/PD-L1 signaling pathway makes a relatively significant contribution to the inhibition of CD8^+^ T cells. Consistent with these observations, the inferred cell-cell communication networks of the PD-L1/PD-L2 signaling pathway showed that CD8_TCs1 were the dominant signaling sources and target at PD-1.

To assess the pivotal role of PD-1 expressing CD8^+^ T cell subsets in conferring long-term protection against tumors, we conducted a rechallenge experiment using MC38-p53^KO/E285K^ tumor models treated with LNP-pE285K-mAb, αPD-1 or their combination. Thirty-five days after tumor rechallenge, the group treated with LNP-pE285K-mAb exhibited a significant reduction in tumor growth compared with the control group (Fig. 5G and H). Treatment with αPD-1 alone also resulted in a substantial reduction in tumor volumes. Notably, a significant enhancement in tumor elimination was observed with the combination of LNP-pE285K-mAb and αPD-1. Monitoring tumor progression for 70 days in the challenge experiment revealed a sustained suppression of tumor growth in the combination group compared with that in naive mice (Fig. 5I). Furthermore, recipients of the LNP-pE285K-mAb treatment showed a 100% survival rate, indicating robust resistance to tumor progression. These results strongly suggested the induction of memory CD8^+^T cell responses by LNP-pE285K-mAb against the p53^E285K^ antigen. To provide anti-tumor immunity, transiently stimulated CD8^+^ T cells undergo unusually rapid bursts of numerous cell divisions and form quiescent, long-lived memory cells that remain poised to reproliferate following subsequent immunological challenges [41]. In this regard, we examined the subsets of memory CD8^+^ T cells (naïve CD44^low^CD62L^high^CD8^+^ T cells, CD8^+^ T_N_; central memory CD44^high^CD62L^high^CD8^+^ T cells, CD8T_CM_; effector memory CD44^high^CD62L^low^CD8^+^ T cells, CD8^+^ T_EM_) 35 days after tumor challenge using FACS analysis based on CD44 and CD62L markers. Interestingly, compared with LNP-E285K-mAb-treated mice, the percentage of CD8^+^ T_N_ was efficiently reduced in the combined treatment group, and no significant difference was observed in CD8^+^ T_CM_, whereas CD8^+^ T_EM_ showed a statistically significant increase in the combination treatment group (Fig. 5J and K). Consequently, the long-lasting protective effect against the tumor could be predominantly attributed to the potent antigen clearance effect mediated by memory CD8^+^ T cell immune responses.

### LNP-pE285K-mAb expands TCR diversity in CD8^+^ T cell anti-tumor responses

**Fig. 6:**
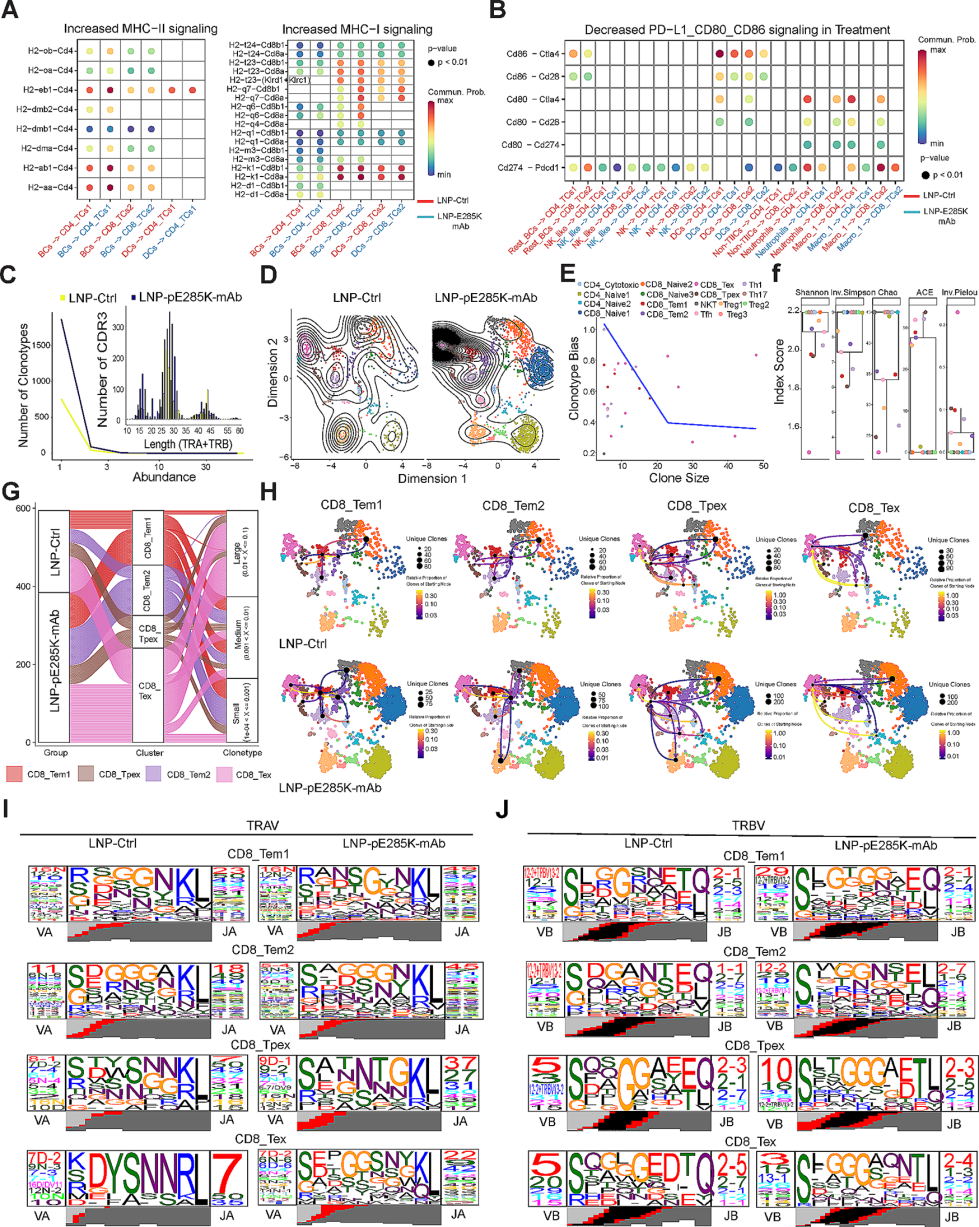
LNP-pE285K-mAb stimulates special TCR motifs for anti-tumor CD8 T cell responses. **A** Enhanced signaling of MHC-I and MHC-II identified by comparing communication probabilities mediated by ligand–receptor pairs from B cells, DCs, to T cell subtypes. **B** Identification of altered ligand–receptor pairs from APCs to CD4^+^ and CD8^+^ T cells by comparing communication probabilities between the two groups. **C** Analysis of total TCR clonotype abundance by sample and type using the abundance contig function. Assessment of CDR3 peptide length by sample using the length contig function. **D** UMAP visualization of TCRs identified in T cells, with clonal overlay using dimensional reduction graphs. **E** Assessment of clonotype bias. **F** TCR clonal diversity. **G** Alluvial plots illustrating the frequencies of TCR clonotypes from each sample, in relationship to the top V(D)J pairing frequencies of expanded clonotypes in each group (right) and contacts (left) among four CD8^+^ T cell clusters. **H** UMAP visualization overlay identifying the network interaction of clonotypes shared between clusters along the single cell dimension reduction. The relative proportion of clones transitioning from a starting node to a different cluster, visualized by arrows in four CD8^+^ T cell cluster networks. **I** and **J** Analysis of TCR sequence motifs of α and β chains for the clonotypes within CD8^+^ T cell clusters by profiles of V and J regions and the CDR3 motif

Efficient clearance of tumors relies heavily on antigen-presenting cells that deliver tumor antigens to activated T cells [42]. We investigated the MHC signaling pathways originating from B cells and DCs to CTL cells using scRNAseq. There was an increased MHC-II signaling (H2-eb1, H2-ab1, and H2-aa) in BCs to CD4_TCs1, an increased MHC-I signaling (H2-q7, H2-q6, H2-k1) from B cells to CD8_TCs2, and an increased MHC-I signaling (H2-q7, H2-k1) and a decreased MHC-II signaling (H2-ab1, H2-dma, H2-dmb-1 and H2-dmb2) from DCs to CD8_TCs2 or CD4_TCs1 upon treatment (Fig. 6A and Supplementary Fig. S8A and B). This result indicates that there is an enhanced MHC antigen presentation from B cells to T cells by the LNP-pE285K-mAb treatment. The interaction of immunosuppressive receptors and their ligands leads to T cell exhaustion and disorder, resulting in a loss of control over tumors [43, 44]. We observed a reduction in PD-L1:PD-1 signaling in NK_like to CD4_TCs1 and CD8_TCs2, NK, neutrophils or macro_1 to CD4_TCs1, and DCs, macro_1 to CD8_TCs2 in the treated group, and a decrease in CD86:CTLA-4 or CD86:CD28 in DCs to CD4_TCs1 and CD8_TCs2 (Fig. 6B). These results indicate that the mAb treatment inhibited the immunosuppressive signaling in APCs, NK, and neutrophils, facilitating T-cell activation against tumor cells.

T cell receptor (TCR) recognizes peptide antigens presented by MHCs from tumor cells or APCs to activate T cells, promoting their division and differentiation [45, 46]. We extracted TCR sequences from scRNA-seq, which revealed a significant increase in the number and abundance of TCRs and their complementary-determining region 3 (CDR3s) in the treated group (Fig. 6C and Supplementary Fig. S8C-F). Clonal overlay using dimensional reduction graphs showed a substantial increase in the number of TCR clones in CD8_Tem1, CD8_Tem2, CD8_Tpex, and CD8_Tex subsets upon treatment (Fig. 6D). TCR clonotype bias and size were the highest in CD8_Tem1 and CD8_Tex clusters (Fig. 6E). TCR clonal diversity was the highest among CD8_Tex, CD8_Tpex, and CD8_Tem1 clusters [36] (Fig. 6F and Supplementary Fig. S8G). Next, we used alluvial plots to analyze the relationships between the top V(D)J pairing frequencies of expanded TCR clonotypes and cell types. Tracking TCR clonotypes based on scTCRseq data revealed an increase in the number and expansion of clonal TCRs of the CD8_Tem1, CD8_Tem2, CD8_Tpex, and CD8_Tex subsets following LNP-pE285K-mAb treatment (Fig. 6G). These scTCR-seq data revealed a visible T cell immune response within tumors from mice treated with LNP-pE285K-mAb, consistent with the scRNA-seq results.

We visualized TCR clonotype network interactions, along with the single-cell dimensional reduction, illustrating the relatively increased proportion of CD8_Tem1 or CD8_Tem2 clones, which transition towards CD8_Tex, CD8_Tpex, and NKT, as indicated by the arrows in the treated group (Fig. 6H). Bidirectional movement of clonotype distribution was depicted, with CD8_Tpex and CD8_Tex showing greater interconnection between clusters through their TCRs. Overlaying the clonal interaction network with UMAP visualized the directionality of the network interactions with the CD8_Naive2, CD8_Tem1, CD8_Tem2, and CD8_Tex clusters (Fig. 6H). CD8_Tem1 and CD8_Tem2 exhibited efflux of the proportion of clones from the starting node in these clusters out to CD8_Tex, CD8_Tpex, and NKT clusters, with 75 and 100 unique clones moving from the starting node to the ending node. Similarly, within the CD8_Naive2 population, clonal expansion proportionality moved inwards towards CD8_Tpex and CD8_Tex, with a large number of 200 unique clones. However, a high proportion of clones started from CD8_Tpex and CD8_Tex and moved into the CD8_Tem1 clusters. Moreover, when compared with the control group, the clusters of CD8_Tem1, CD8_Tem2, CD8_Tpex, and CD8_Tex exhibited distinct specificity in epitopes within TCR sequences. These clusters showed biases in TRAV or TRBV-segment usage and displayed a strong preference for specific amino acid residues at distinct positions of the CDR3 region of the treated group (Fig. 6I and J). These results indicate that LNP-pE285K-mAb promotes the enrichment of specific CDR3 motifs and enhances CD8^+^ T cell responses.

The same CD45^+^ cell pool was subjected to B cell receptor (BCR) sequencing. A substantial increase in the number of BCR clones was observed in the treated group (Supplementary Fig. S9A and B). Total abundance, space occupied, CDR3 length, and diversity of BCR clonotypes also increased (Supplementary Fig. S9C-F, and H). The clonotypes of dominant CDR3 sequences were reduced in B cells after treatment (Supplementary Fig. S9G and H). The clonotypes across diversity and V(D)J pairing frequencies in B and B-like cell subsets showed that B cells were the dominant class (Supplementary Fig. S9I-K). These results indicated that LNP-pE285K-mAb changes BCR profiles in tumor-infiltrating B cells.

### The anti-tumor effect of LNP-pE285K-mAb requires TRIM21 in tumor cells

**Fig. 7:**
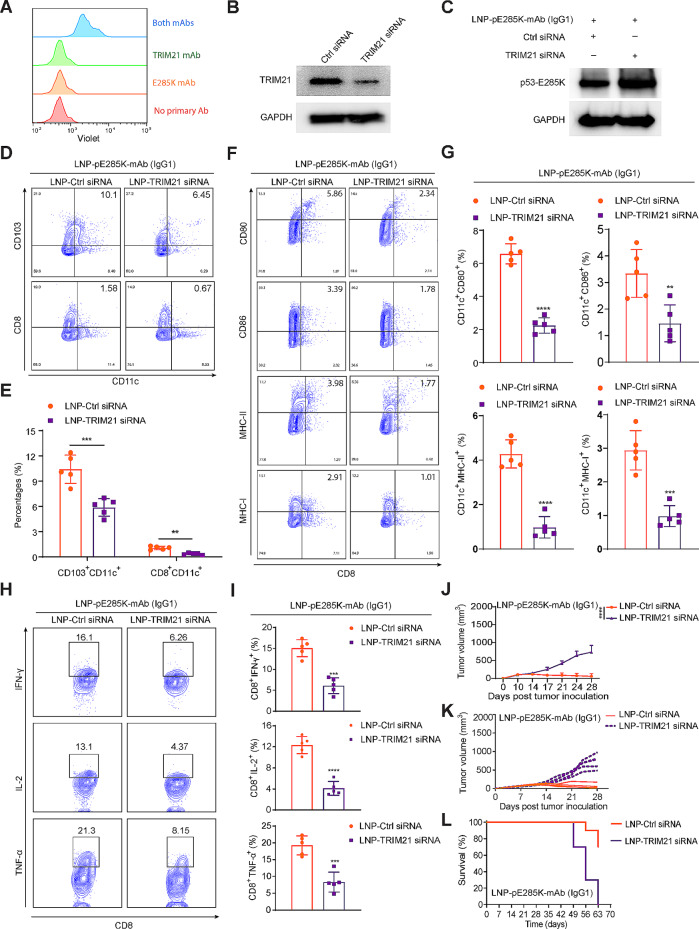
The anti-tumor effect induced by E285K-mAb in the IgG1 format requires TRIM21. **A** Protein interaction between TRIM21 and E285K-mAb assayed by FACS in MC38-p53^KO/E285K^ cell treated with E285K-mAb and TRIM21-mAb. **B** and **C** Detection of TRIM21 knockdown efficiency and p53-E285K expression levels post siRNA treatment in MC38-p53^KO/E285K^ cells by western blot. **D** and **E** Analysis of CD103^+^CD11c^+^ and CD8^+^CD11c^+^ cell percentages in tumors from mice treated with LNP-TRIM21 siRNA and LNP-pE285K-mAb (*n* = 5 mice). **F** and **G** The expression of activation markers (CD80, CD86, and MHC) on CD11c^+^ cells within tumors using FACS. **H** and **I** IL-2, IFN-γ, and TNF-α expression on CD8^+^ T cells. **J** Tumor volumes measured at different times after inoculation. **K** Tumor diameter of individual mice from the groups in (**j**) as a function of time. Each group consisted of five tested mice. **L** Animal survival (*n* = 10 mice). Data presented as means ± SD. Statistical significance was set at **p < 0.01, ***p < 0.001, and ****p < 0.0001

TRIM21 is an intracellular IgG receptor that utilizes a non-Ig scaffold with evolutionarily conserved structural, thermodynamic, and kinetic levels [47]. A recent publication showed that TRIM21 directly interacts with p53^R175H^ but not WT p53, leading to ubiquitination and subsequent degradation of mutant p53 and suppressing tumor development [48]. We performed a PLA using FACS to assess the interaction between TRIM21 and E285K-mAb. TRIM21 and E285K-mAb interacted in MC38-p53^KO/E285K^ cells (Fig. 7A). Next, we knocked TRIM21 down (Fig. 7B), which caused p53^E285K^ upregulation in MC38-p53^KO/E285K^ cells transfected with LNP-E285K-mAb (Fig. 7C). Next, we evaluated tumor development of MC38-p53^KO/E285K^ cells with TRIM21 downregulation in mice treated with a two-dose intratumoral injection of LNP-pE285K-mAb. The percentages of antigen-induced CD103^+^CD11c^+^ and CD8^+^CD11c^+^ DCs in tumors were significantly decreased (Fig. 7D and E). The expression of surface-activated markers (CD80, CD86, MHC-II, or MHC-I) on DCs was inhibited, indicating weakened antigen presentation following TRIM21 knockdown (Fig. 7F and G). This was accompanied by a decrease in the induction and proportion of tumor-infiltrating multifunctional CD8^+^ T cells expressing IFN-γ, TNF-α, or IL-2 (Fig. 7H and I). Finally, Trim21 knockdown inhibited the therapeutic effect of LNP-pE285K-mAb, as evidenced by tumor volumes (Fig, 7 J and K) and animal survival (Fig. 7L). These data suggest that TRIM21 is involved in E285K-mAb activity against tumor cells expressing p53^E285K^.

### The dIgA subtype of E285K-mAb exerts potent anti-tumor effects via PIGR

**Fig. 8:**
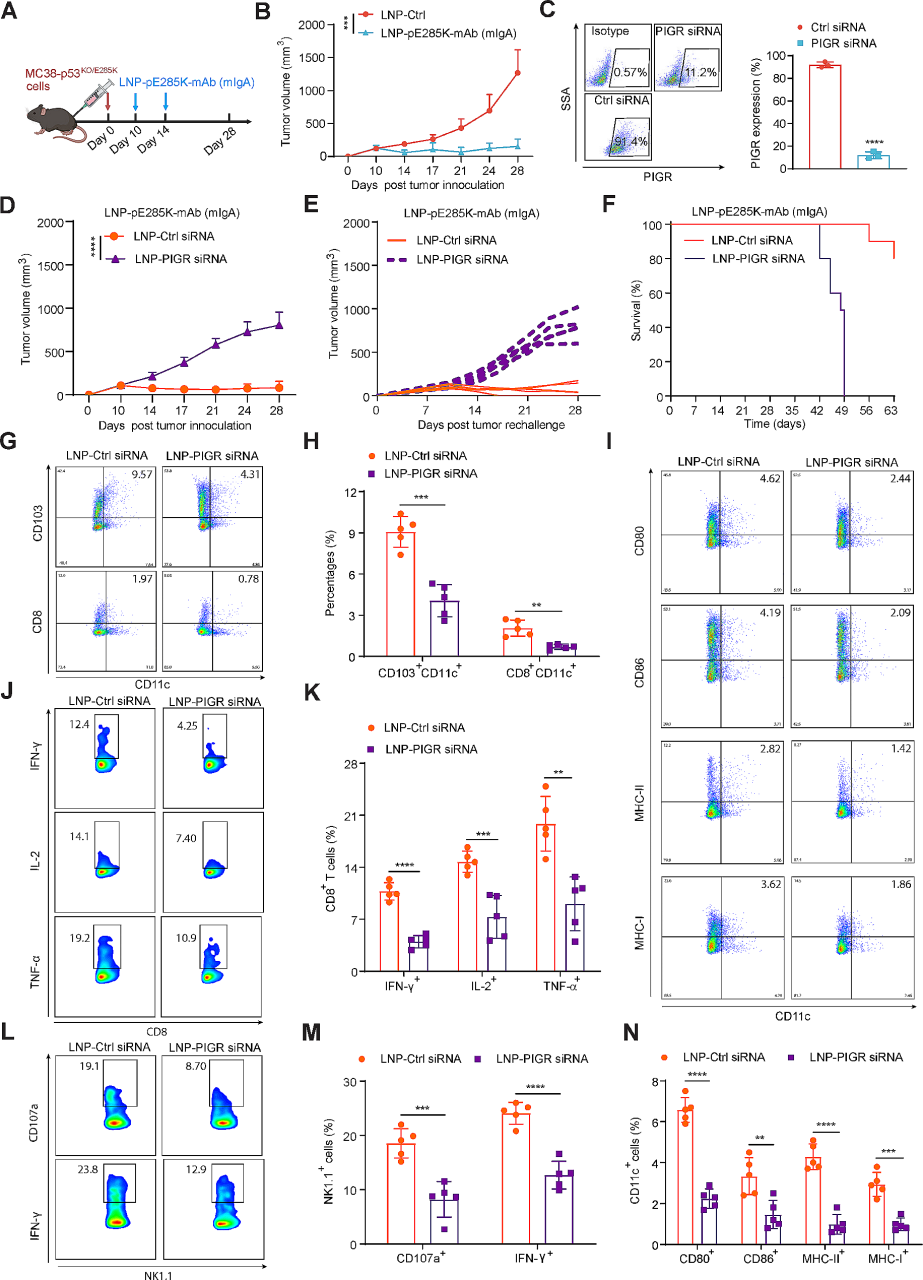
The anti-tumor effect of E285K-mAb in the dIgA format via PIGR. **A** Schema of the subcutaneous MC38-p53^KO/E285K^ tumor model by intratumoral injection of LNP-pE285K-mAb in mouse dIgA isotype (mIgA) on days 10 and 14 post tumor inoculation (*n* = 5 mice). **B** Tumor volumes. **C** Assessment of PIGR expression in MC38 cells treated with PIGR siRNA or control siRNA. **D** Tumor volumes in mice treated with mIgA and LNP-PIGR siRNA (*n* = 5 mice per group). **E** Individual tumor volumes over time in mice specified in (D). **F** Animal survival (*n* = 10 mice per group). **G** and **H** Percentages of DC subsets (CD103^+^CD11c^+^, CD8^+^CD11c^+^) within TILs of tumors treated with LNP-PIGR-siRNA. **I** and **N** CD80, CD86, and MHC expression on CD11c^+^ cells within TILs. **J** and **K** IL-2, IFN-γ, and TNF-α expression on CD8^+^ T cells within TILs. **L** and **M** CD107a and IFN-γ expression in NK cells within TILs. Data were presented as means ± SD. Statistical significance was set at **p < 0.01, ***p < 0.001, and ****p < 0.0001

Tumor-infiltrating B cell-derived dIgA redirects myeloid cells against extracellular oncogenic drivers, such as KRAS^G12D^, causing tumor cell death [49, 50]. We prepared an mAb against p53^E285K^ in the dIgA format encoded in DNA plasmids and delivered it using LNPs (LNP-pE285K-dIgA). We treated mice with MC38-p53^KO/E285K^ tumors on days 10 and 14 post-tumor inoculation with two doses of LNP-pE285K-dIgA (Fig. 8A). Treatment caused a significant reduction in tumor volume (Fig. 8B). dIgA promotes the presentation of intracellular neoantigen through PIGR expressed on the tumor cell surface [49, 51]. We knocked down *Pigr* in MC38 tumor cells (Fig. 8C). Next, we delivered anti-Pigr siRNAs via LNPs through intratumoral injection. Pigr knockdown partially blocked tumor inhibition mediated by LNP-pE285K-dIgA (Fig. 8D-F). These data indicated that the therapeutic effect of LNP-pE285K-dIgA is PIGR-dependent. Pigr knockdown reduced the numbers of two DC subsets (CD103^+^CD11c^+^ and CD8^+^CD11c^+^) within tumors (Fig. 8G and H) and down-regulated the co-stimulatory molecules CD80 and CD86 and the antigen-presenting molecules MHC-II and MHC-I (Fig. 8I and N). Consequently, the induction of IFN-γ, TNF-α, and IL-2 in tumor-infiltrating CD8^+^ T cells by LNP-pE285K-dIgA was inhibited upon Pigr knockdown (Fig. 8J and K). The percentage of NK cells expressing CD107a and IFN-γ was also reduced (Fig. 7L and M). These data support the hypothesis that PIGR is critical to the therapeutic activity of the dIgA antibody.

## Discussion

Most cancer therapeutic mAbs are in IgG format to specifically bind to antigens on the cell surface or to circulating cytokines and chemokines. Once bound, mAbs guide the immune system to attack tumor cells by activating NK and cytotoxic T cells, guiding macrophages for phagocytosis, or inducing cell apoptosis and other pathways [52, 53]. The production and purification of high-quality IgG typically involve intricate processes, resulting in higher production costs. In certain instances, exogenous IgG may elicit reactions in the immune system, including antibody generation and rejection responses [54, 55]. Limited studies have targeted intracellular oncoproteins using mAbs.

In this study, we developed specific mAbs directed against the mutant p53^E285K^ antigen, exhibiting no cross-reactivity with wild-type p53 or other mutants like p53^R282W^. The intratumoral delivery of tumor tissue-targeted mAbs increases the in-situ bioavailability and efficacy of immunotherapies with minimal toxicities [56]. We used the LNP system from the Moderna COVID mRNA vaccine to carry DNA plasmids encoding IgG1 mAb for intratumoral delivery. The IgG1 subtype mAb has potent neutralizing activity and enhances the clearance capacity against target cells with a longer half-life, providing prolonged immune protection [57]. Due to the intracellular localization of the p53^E285K^ protein in tumor cells, E285-mAb displays a diminished in vitro ADCC effect. However, the LNP-pE285K-mAb is more potent than E285K-mAb, primarily because of the reduced presentation of membrane surface antigen peptides, posing a challenge for mAb to target tumors. In this study, we demonstrated that E285K-mAb can bind to TRIM21. Therefore, within tumor cells, E285K-mAb can selectively bind to the p53^E285K^ antigen, and the TRIM21 bound by E285K-mAb is then brought to the p53 antigen. This leads to the enrichment of TRIM21 around p53^E285K^, facilitating ubiquitination-mediated degradation. In the MC38-p53^KO/E285K^ tumor mouse model, administration of LNP-pE285K-mAb treatment only twice markedly suppressed tumor growth, achieving close to 40% CR and 100% survival rates. Safety assessments show no observable inflammatory reactions in vital organs, such as the heart, liver, spleen, lungs, and kidneys, in LNP-pE285K-mAb-treated mice. Serum inflammatory cytokines only transiently increased and returned to normal after five days, indicating the excellent low-toxicity safety of intratumoral LNP-mediated treatment. To comprehensively investigate the effects of this LNP-delivered E285K-mAb, we established colorectal cancer in situ models, lung metastasis models, and humanized mouse models of lymphoma or breast cancer. LNP-pE285K-mAb effectively inhibits the growth and metastasis of these tumors, demonstrating an excellent preclinical therapeutic effect and laying the foundation for the following clinical assessment.

Our FACS, scRNAseq, and antibody blocking data indicated that intratumoral administration of LNP-pE285K-mAb markedly enhanced the infiltration of immune cells, including B cells, B-like cells, IFIT^+^_BCs, CD4^+^ T, CD8^+^ T, and NKT cells, while concurrently reducing the infiltration of tumor-associated macrophages, Rest_BCs, and neutrophils in MC38-p53^KO/E285K^ subcutaneous tumors. The outgoing and incoming signals from IFIT^+^_BCs and B-like cells to CD8_TCs1 and CD8_TCs2 are persistently strengthened, aligning with the established role of B cells in mediating CD8^+^ T cell immune responses in tumor control. Cd74^+^NK_like cells exhibit a significant increase in the treated group. These cells exhibit both B cell and NK cell functions. B or NK cell depletion by blocking antibodies markedly nullified the therapeutic effects of LNP-pE285K-mAb, supporting the pivotal role of these two cell types. The LNP-pE285K-mAb treatment markedly increased the number of tumor-infiltrating CD8_Naive, CD8_Tem, and CD8_Tex populations, supporting the anti-tumor activities of memory and exhausted CD8^+^ cytotoxic T cells. The treatment also upregulates Pdcd1 expression and other checkpoint molecules, and PD-1 blockade enhanced the efficacy and persistence of the anti-tumor effects by increasing the proportion and intensity of induced memory CD8^+^ T cells. We noted that the quantity and abundance of TCRs in treatment induced CD8_Tem, CD8_Tpex, and CD8_Tex cells increased significantly, as did the expansion of specific CDR3 motifs. TRIM21 is a cytosolic ubiquitin ligase and antibody receptor that participates in intracellular antibody-mediated proteolysis. Trim21 knockdown leads to a higher level of the p53^E285K^ protein and reduces the anti-tumor activity of the LNP-pE285K-mAb. It is likely that TRIM21 participates in the degradation of p53^E285K^ with the help from E285K-mAb to produce antigenic peptides for APC presentation. It is reported that TRIM21 directly binds several p53 mutants (R175H, G245S, R248Q, and R273H) [48]. However, we do not have evidence to support that, without E285K-mAb, TRIM21 binds to p53^E285K^.

dIgAs against KRAS^G12D^ and IDH1^R132H^ promote antigen expulsion from the cytosol of PIGR^+^ tumor cells and impede tumor growth [51]. We constructed an IgA subtype targeting p53^E285K^ that exhibited a substantial inhibitory effect on tumor development upon LNP-mediated delivery. We confirmed that the IgA subtype E285K antibody acts through the PIGR receptor on the surface of tumor cells. These translocated antigens are likely captured by DC cells, activating antigen specific CD8^+^ T cell immune responses and effectively inhibiting the growth and development of tumors.

Mouse p53 was discovered as a cellular tumor antigen in 1979 [58–60]. Serum antibodies against human mutant p53 have been found in cancer patients since 1982 [61, 62], yet are not commonly associated with better survival [62, 63]. Tumor-infiltrating B cells and plasma cells (TIL-Bs), as part of TIICs, from cancer patients also express antibodies targeting p53 [64–69], and TIL-Bs are associated with better survival in most cancers [64]. Mounting evidence has shown that TIL-Bs play a crucial and multifaceted role in tumor control [64]. TIL-B-derived antibodies and serum-derived autoantibodies against most antigens may originate and persist independently in cancer patients [64, 70]. Recent evidence supports that TIL-B-derived antibodies are produced locally within tertiary lymphoid structures that arise de novo in hot tumors [64, 65]. Overall, serum-derived and TIL-B-derived antibodies support that cancer patients have humoral immune responses against mutant p53, underlying the scientific premise to develop personalized mAbs that are specific to the p53 mutant epitopes. TIL-Bs promote antitumor immunity in most cancers through cell- and antibody-based effector mechanisms [64, 70]. Leveraging the antibody-mediated effects of TIL-Bs has been proposed as safe and effective cancer therapies [64]. Most studies suggest that IgG but not IgA from TIL-Bs is associated with better prognosis [64], but others show IgA is more pro-survival [49]. This study is the first to report the development of both IgG1 and dIgA mAbs specific to a mutant p53 epitope, attenuating the growth of tumor cells carrying the p53 mutant. The IgG1 and dIgA represent the antibody effector function of TIL-Bs, albeit engineered, optimized, and amplified for maximum potency and minimal autoimmune responses by not targeting WT p53.

## Conclusions

Our study elucidates the potent therapeutic effects of IgG1 and dIgA targeting p53^E285K^ in attenuating tumor development and metastasis. These findings underscore the therapeutic efficacy of LNP-mediated DNA-encoded mAb delivery and offer a novel strategy for treating cancers with intracellular oncoproteins. However, this study possesses several limitations. First, it uses one murine cell line and two human cell lines as proof of concept, and more preclinical investigations should corroborate to unlock the translational potential. Some mutant p53 epitopes may remain inaccessible to antibody targeting due to the formation of aggregates by many mutant p53 proteins. Further studies are needed to dissect the underlying mechanisms of IgG1 and dIgA against p53 mutants in target engagement, immune activation, and tumor inhibition.

### Electronic supplementary material

Below is the link to the electronic supplementary material.

Supplementary Material 1

Supplementary Material 2

## Data Availability

All the data that support the findings of this study are available within the article and supplemental information or available from the authors upon request. The original RNA-seq data was deposited to Gene Expression Omnibus (GEO) and can be accessed using the GEO series accession code GSE260908.

## Abbreviations

mAbs: monoclonal antibodies
LNPs: Lipid nanoparticles
Fc: Fragment crystallizable region
Fab: Fragment antigen-binding region
scRNA-seq: single-cell sequencing
dIgA: dimeric IgA
PIGR: Polymeric immunoglobulin receptor
WT p53: Wild-type p53
TME: Tumor microenvironment
BLI: Bio-Layer Interferometry
PBMCs: Peripheral blood mononuclear cells
TIICs: Tumor-infiltrating immune cells
TCR: T cell receptor
