# Supplementary material for: Remodeling of anti-tumor immunity with antibodies targeting a p53 mutant
Source: J Hematol Oncol. 2024 Jun 18;17:45. doi: 10.1186/s13045-024-01566-1 (PMC11184848; doi:10.1186/s13045-024-01566-1)
Supplement: Supplementary file 1 — Supplementary Material 1 [file 13045_2024_1566_MOESM1_ESM.pdf]

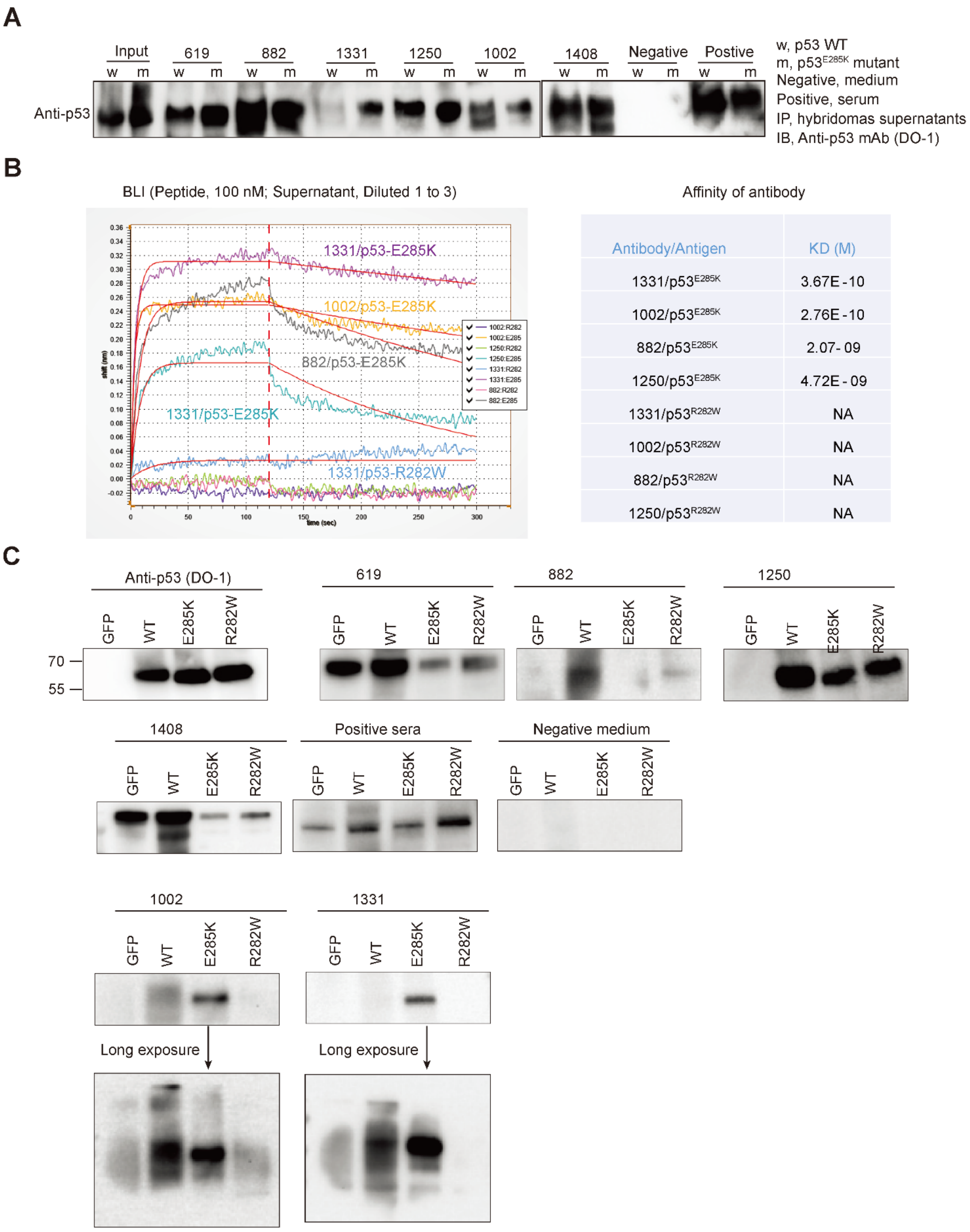

Fig. S1 Screening and characterization of E285K-mAb. A Assessment of six E285K-mAb clones for antigen binding by immunoprecipitation. Hybridomas supernatants containing E285K-mAbs (clone 619, 882, 1331, 1250, 1002, and 1408) were incubated with WT p53 or p53^{E285K} antigen, followed by immunoprecipitation and

western blotting detection using anti-p53 mAb (DO-1 targeting both WT and p53 mutants). **B** BLI kinetics of four E285K-mAbs binding to the $p53^{E285K}$ or a control peptide ($p53^{R282W}$). **C** Analysis of WT p53 or $p53^{E285K}$ expression in 293T cells transfected with WT p53, $p53^{E285K}$, $p53^{R282W}$, or the GFP control by western blot assay using six E285K-mAb hybridoma supernatants.

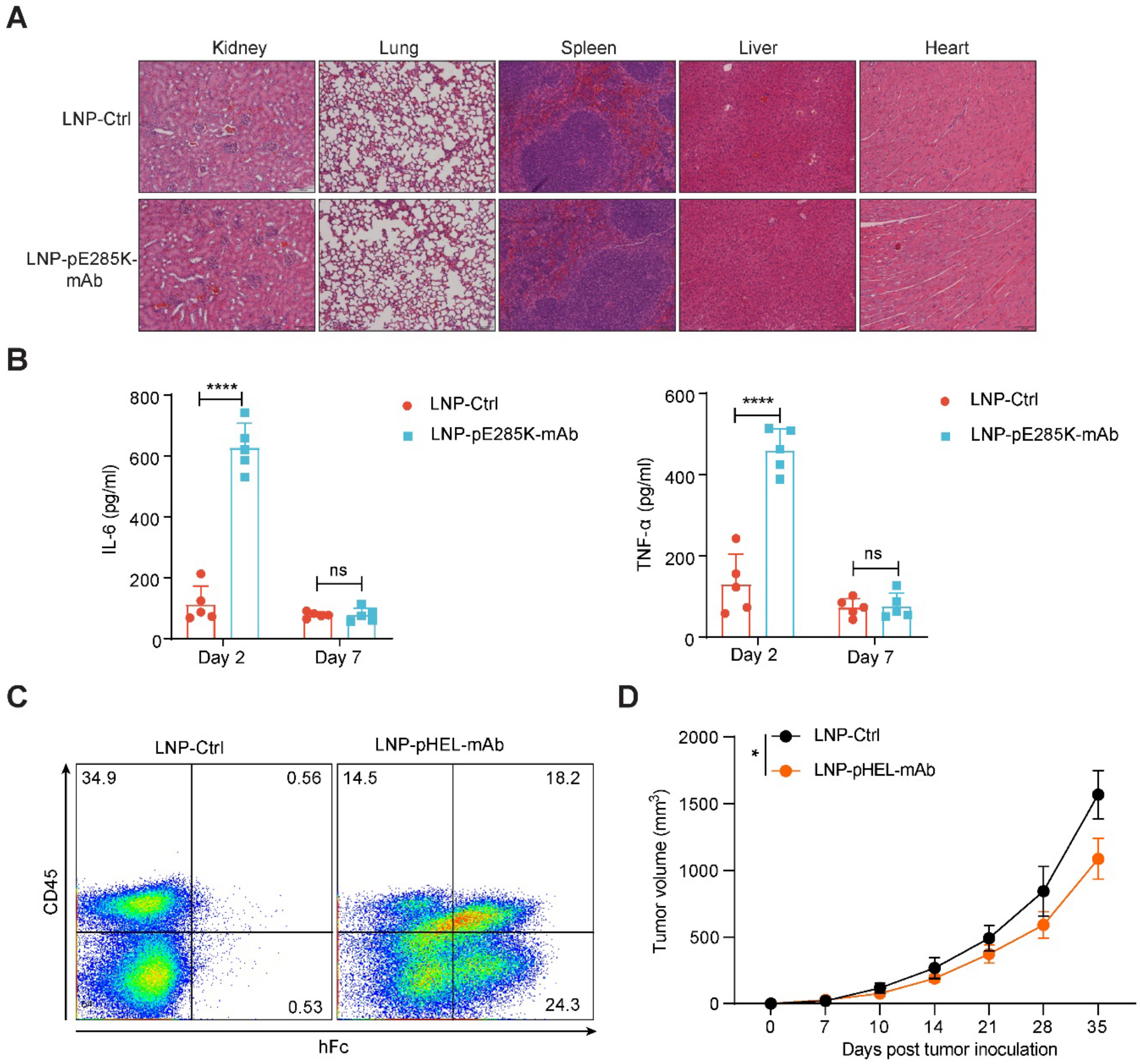

Fig. S2 Safety evaluation of LNP-pE285K-mAb in mice with subcutaneous MC38-$p53^{KO/E285K}$ tumors. A Histopathological examination of kidney, lung, spleen, liver, and heart tissues via H&E staining in mice treated with LNP-pE285K-mAb or LNP-Ctrl. **B** Measurement of serum IL-6 and TNF-α levels by ELISA in mice treated with LNP-pE285K-mAb or LNP-Ctrl. **C** Detection of HEL-mAb expression and binding in tumors treated with LNP-pHEL-mAb or control using anti-hFc mAb by FACS. **D** No anti-tumor activity from the control HEL-mAb. Mice were subcutaneously inoculated with 3×10^5 MC38-$p53^{KO/E285K}$ cells on day 0 and

intratumorally injected with LNP-pHEL-mAb or LNP control. Tumor progression was evaluated by measuring tumor volumes once every 7 days from day 0 to day 35 (n=5 mice). Data were represented as means ± SD. Statistical significance was set at *p < 0.05.

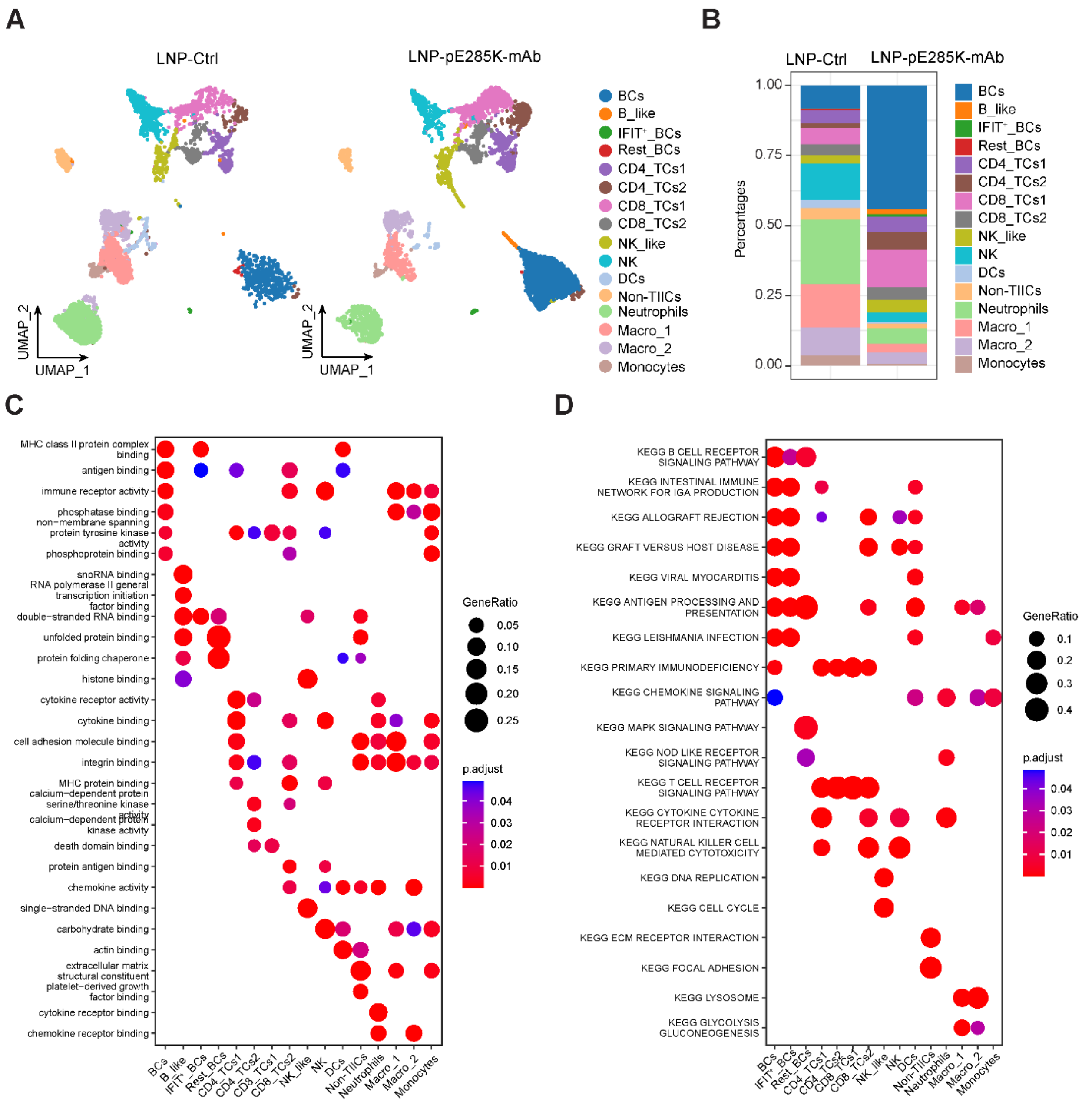

Fig. S3 Characteristics of transcriptomic clustering of immune cells. A Visualization of immune cell landscape by scRNA-seq in LNP-Ctrl and LNP-pE285K-mAb groups, featuring 16 distinct cell types, depicted in two separate UMAPs. **B** Proportion and frequency of each cell type in two groups. **C** and **D** Gene set enrichment analysis of immune cell types using GO gene sets (c) and KEGG gene sets (d), with pathway enrichment expressed as gene ratio.

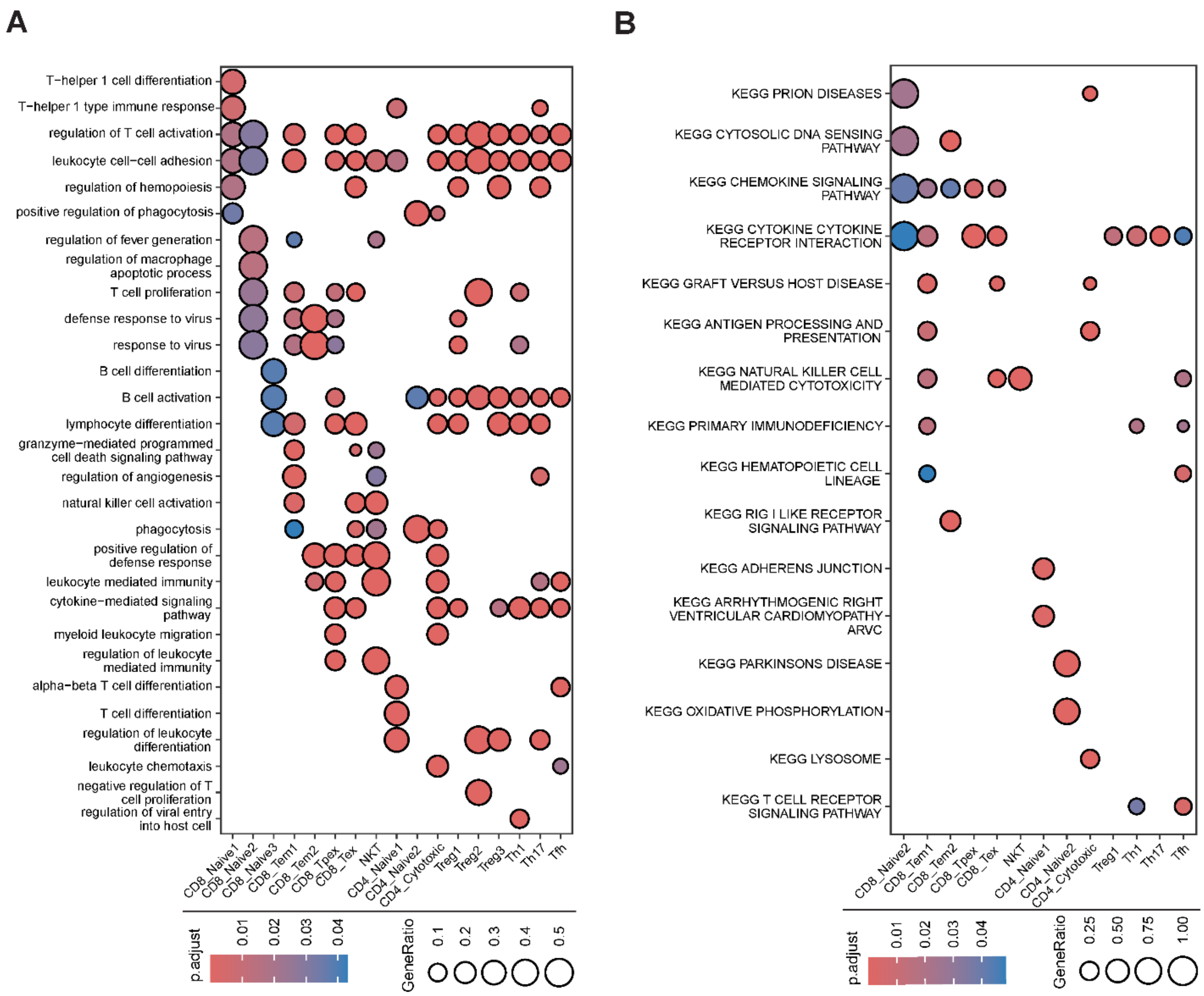

Fig. S4 Pathway enrichment of T cell subsets. A and **B** Gene set enrichment analysis of T cell subsets using GO gene sets (**A**) and KEGG gene sets (**B**). Pathway enrichment is quantified as gene ratio.

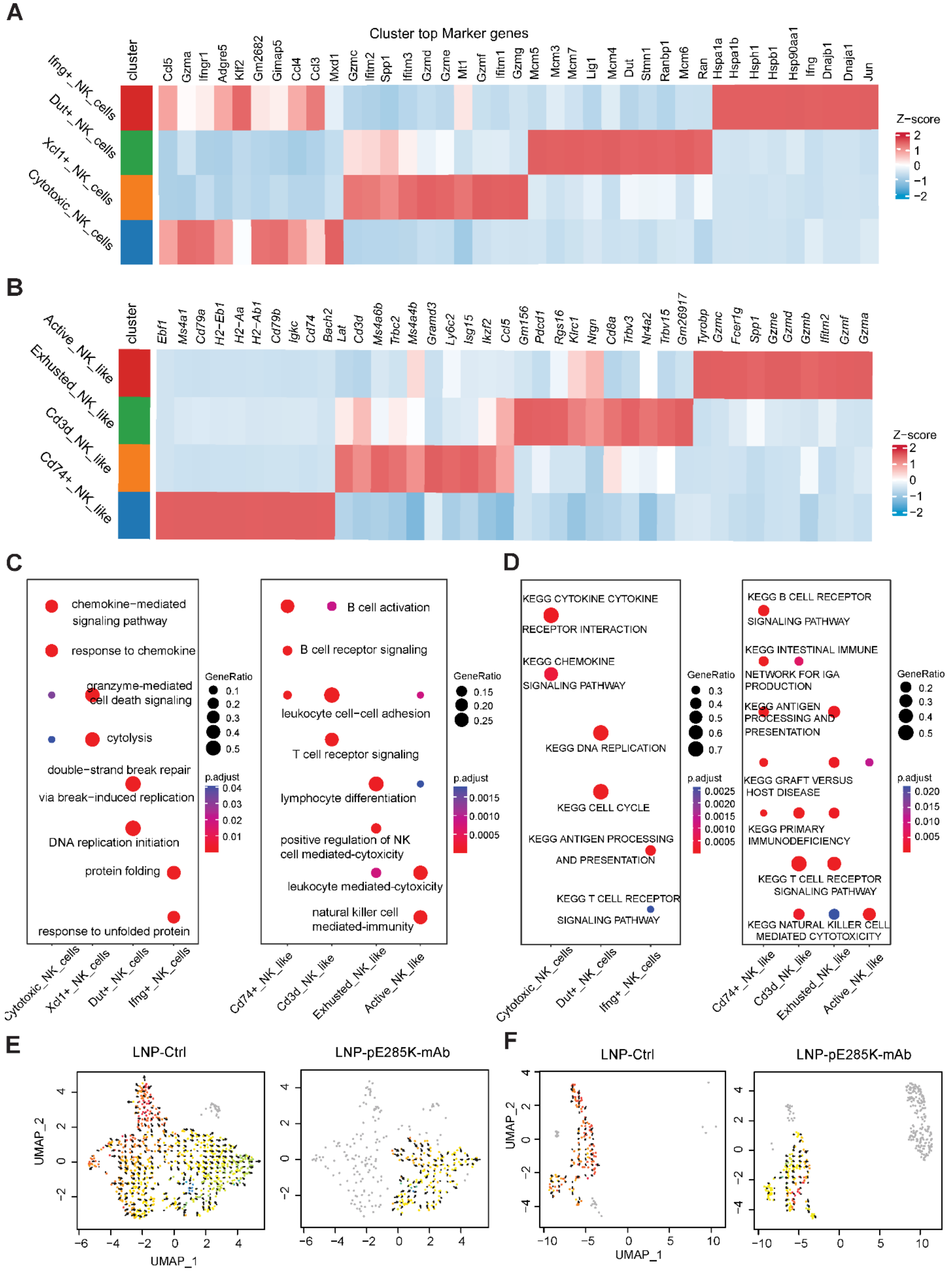

Fig. S5 Pathway enrichment and developmental inference analysis of transcriptomic clustering of NK and NK-like cells. A and **B** Heatmap showing the expression level of top markers in each NK and NK-like cell

subcluster. **C** and **D** Pathway enrichments of top DEGs in NK and NK-like cell subsets using GO gene sets (**C**) and KEGG gene sets (**D**). Pathway enrichment is expressed as gene ratio. **E** and **F** Developmental inference analysis shows the dynamic shift in the state of NK (**E**) and NK-like (**F**) cell subsets from two groups. Arrow predicates the direction of cell state transition.

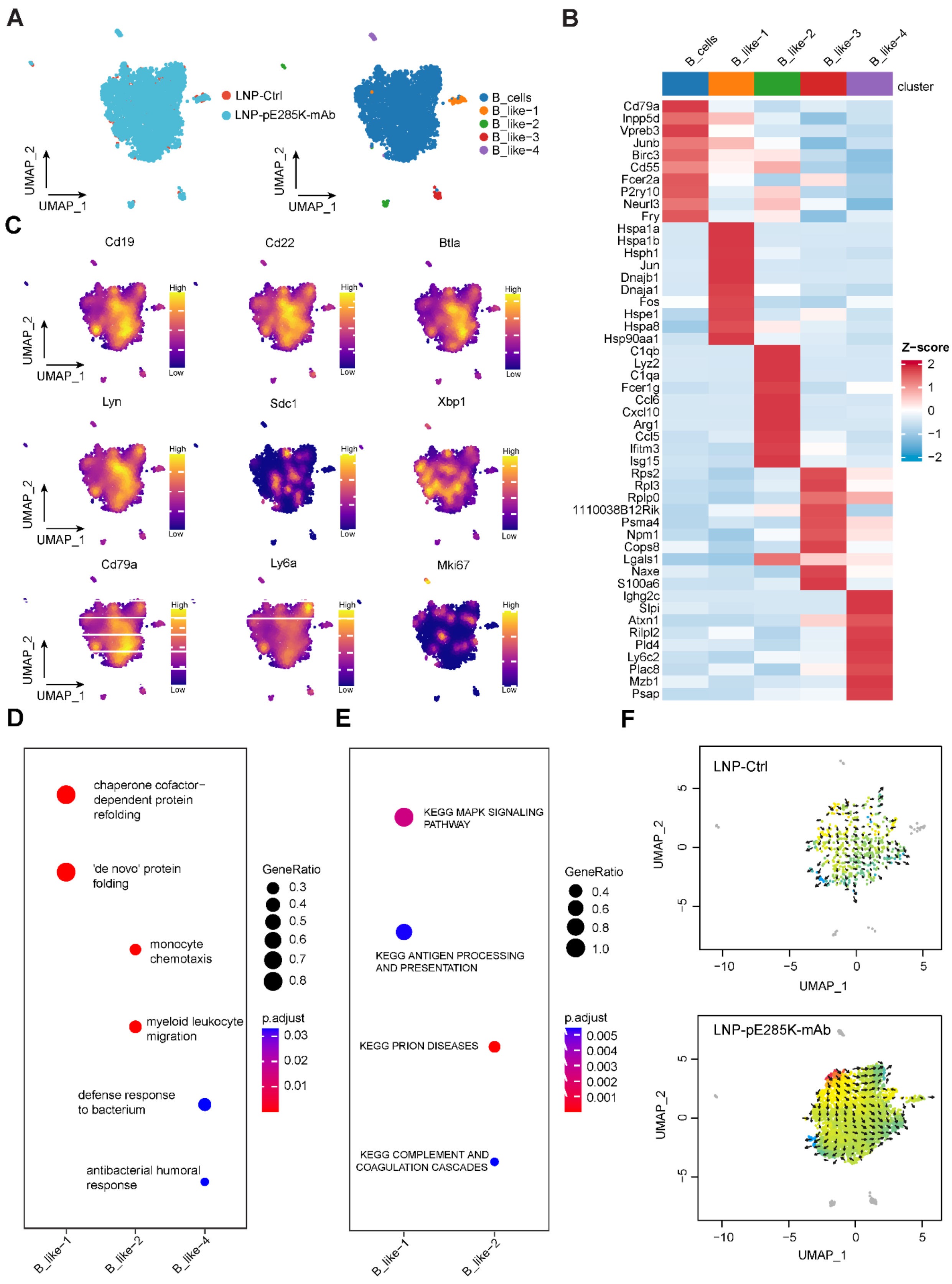

Fig. S6 Characteristics of transcriptomic clustering of B cells. **A** UMAP projection of five B cell clusters from tumors of LNP-pE285K-mAb and LNP-Ctrl-treated mice. Each dot represented a single cell, and each

color represented a cell cluster. **B** Heatmap showing the average expression levels and cell expression intensity of top DEGs in the indicated five B cell clusters. **C** Density plots comparing the expression levels of representative markers in B cell subtypes. **D** and **E** Pathway enrichments of top DEGs in B cell subsets using GO gene sets (**D**) and KEGG gene sets (**E**). Pathway enrichment is expressed as gene ratio. **F** Developmental inference analysis shows the dynamic shift in the state of B cell subsets from two groups. Arrow predicates the direction of cell state transition.

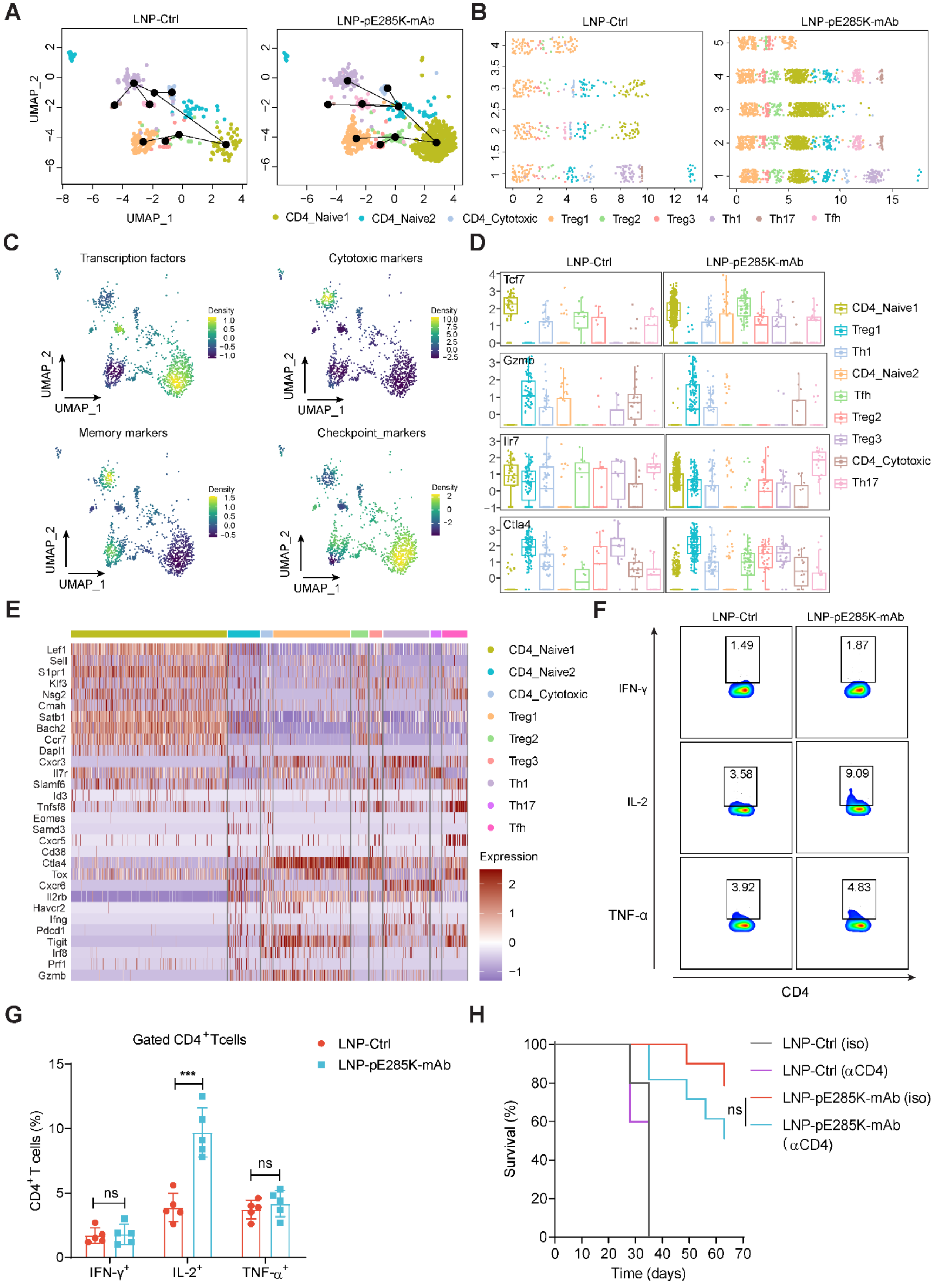

Fig. S7 The therapeutic effect of LNP-pE285K-mAb is independent of CD4^{+} T cells. **A** UMAP showing the

developmental trajectory of $CD4^+$ T cell subtypes. **B** Pseudotime analysis of the indicated cell states from (**A**). **C** Number and intensity of different marker expressions from (**A**) projected onto UMAP. **D** Boxplots showing the measurement of the expression of markers by Kruskal-Wallis test. **E** Heatmap of selected top DEGs in several color-coded $CD4^+$ T cell subtypes. **F** and **G** The expression of TNF-α, IL-2, and IFN-γ in $CD4^+$ T cells from TILs detected by FACS. Experiments were performed with five tumors from each group with one representative image. **H** Animal survival. Mice (n=10 per group) were injected intraperitoneally with 0.5 mg/mouse anti-mouse CD4 mAb on day 2 before the first dose of LNP-pE285K-mAb, with the 2nd and 3rd doses on days 5 and 12. Data were represented as means ± SD. Statistical significance was set at ***$p < 0.001$, ns, no significant.

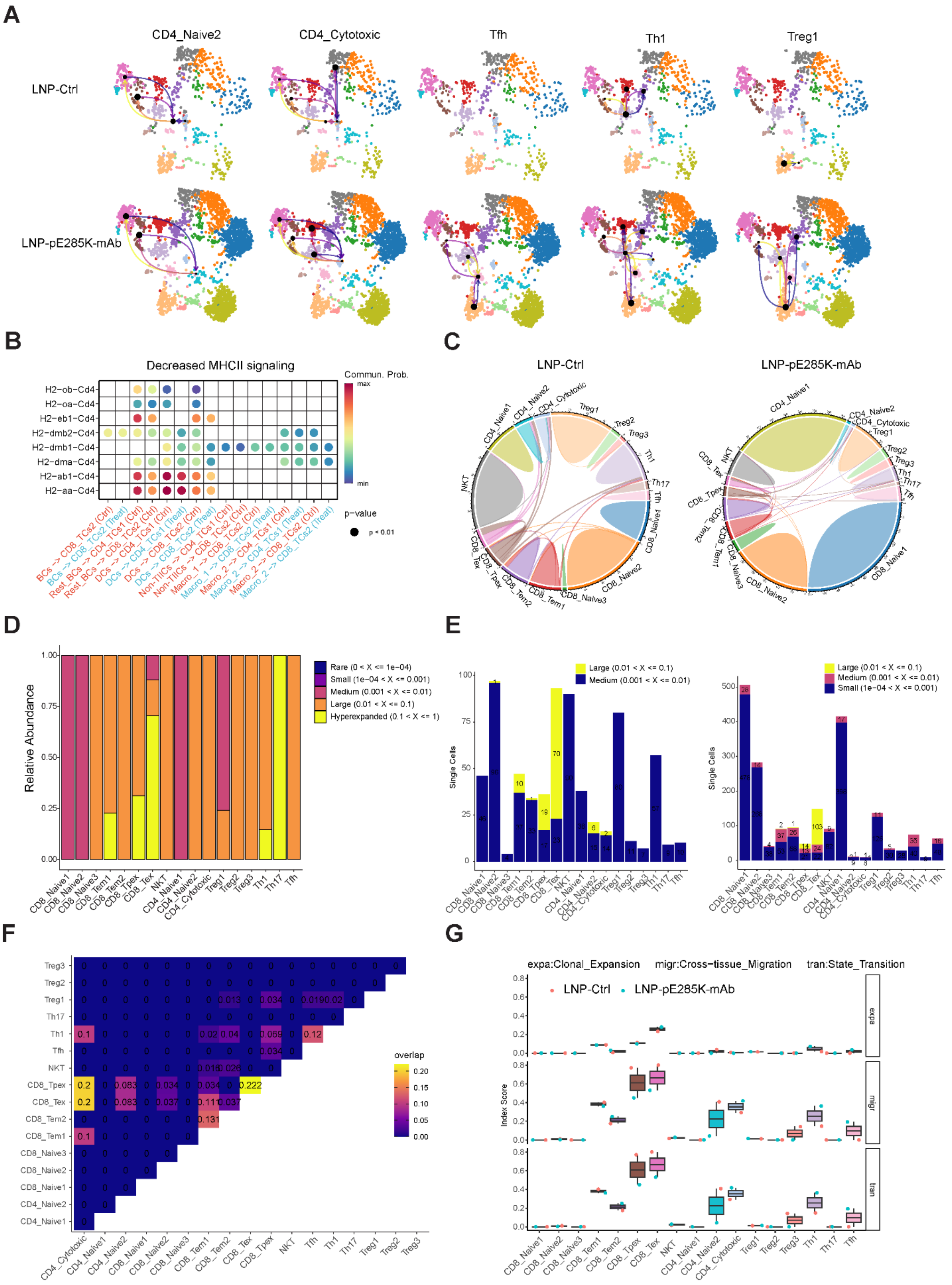

Fig. S8 TCR characteristic of CD4^{+} T cell subsets. **A** UMAP visualization overlay to identify the network interaction of clonotypes shared between clusters along the single cell dimension reduction. Relative

proportions of clones that came from a starting node and end in a different CD4$^+$ T cell cluster were visualized by the arrows. **B** Identification of decreased MHC-II signaling by comparing the communication probabilities mediated by ligand–receptor pairs from B cells, DCs, or macrophages to T cell subsets. **C** Chord diagrams showed the number of relative TCR clonotypes and shared clonotypes across T cell subsets in two groups. **D** Clonal homeostatic space representations (clonal space occupied by clonotypes of specific proportions) of TCRs across T cell subsets. **E** Bar plots showed the number of single cells detected TCR VDJ by T cell subsets from two groups. **F** Morisita overlap quantifications for clonotypes across all T cell subsets. **G** Box plot showing clonotype diversity of 17 T cell subpopulations. Clonotype diversity was calculated as the clonal expansion index, cross-tissue migration index or state transition index within T cell subpopulations.

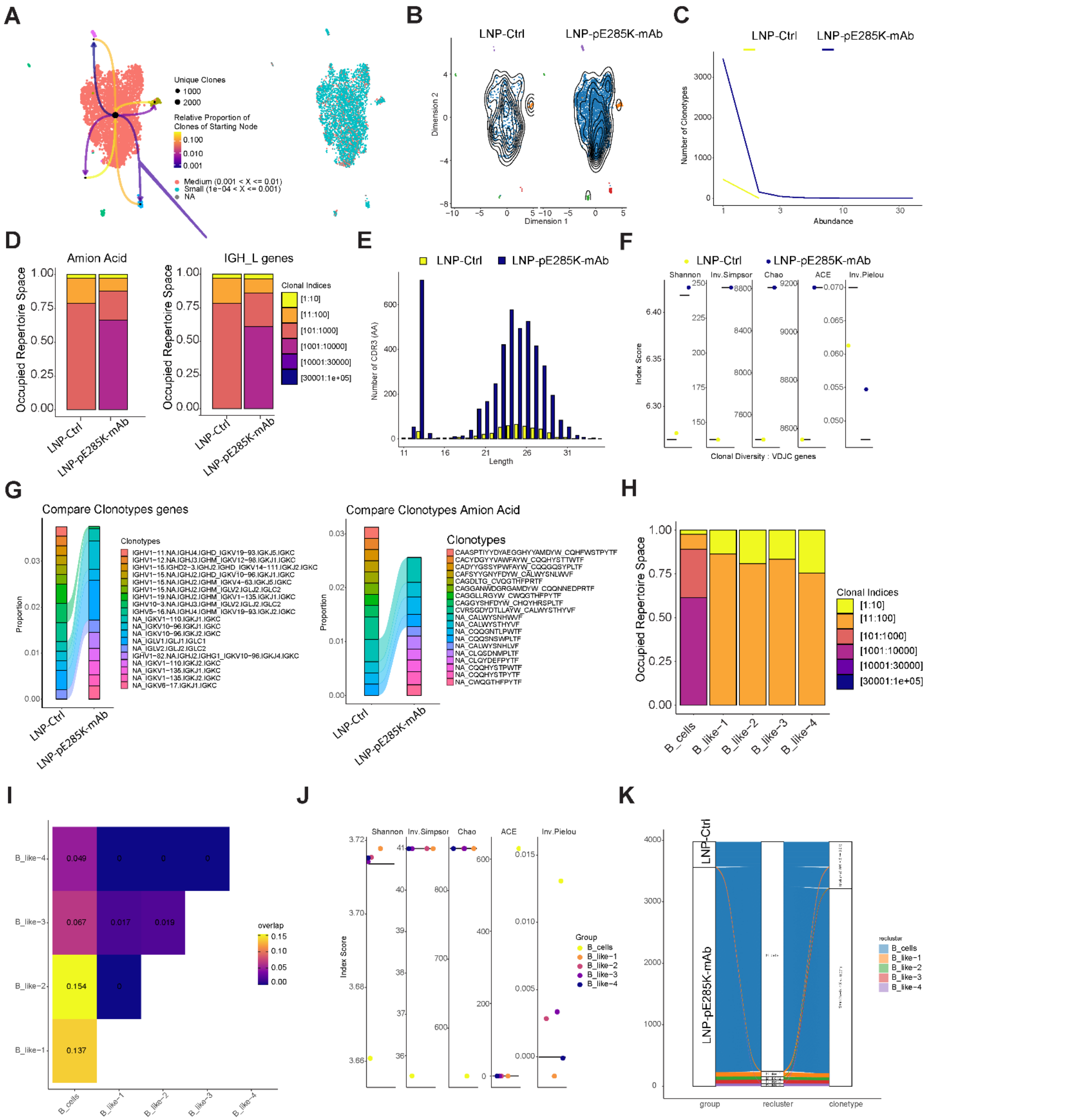

Fig. S9 BCR characteristic of B cell subsets induced by LNP-pE285K-mAb treatment. A UMAP visualization overlay identifying the network interaction of clonotypes shared between clusters along the single cell dimension reduction. Relative proportions of clones that came from a starting node and ended in a different B cell cluster were visualized by the arrows. **B** UMAP visualization of BCRs identified in B cells from two

groups with clonal overlay using the dimensional reduction graphs. **C** Total abundance of B cell clonotypes by sample and type using the abundance contig function. **D** Clonal homeostatic space representations, the relative proportional space occupied by specific clonotypes of BCR (Amino acid and IGH_L genes) across samples. **E** CDR3 length analysis of BCR by samples using the length contig function. **F** Box plot showing clonotype diversity of VDJC genes. Clonotype diversity was calculated as the Shannon index, inverse Simpson index, or Chao index within B cells. **G** Dynamics of dominant clonotype sequences (genes and amino acids) of BCRs across samples, colored by the types of dominant sequences. **H** The relative proportional space occupied by specific clonotypes of BCR across B cell subsets. **I** Morisita overlap quantifications for clonotypes across all B cell subsets. **J** Box plot showing clonotype diversity of VDJC genes. Clonotype diversity was calculated as the Shannon index, inverse Simpson index, or Chao index within B cell subsets. **K** Alluvial plots tracking the frequencies of BCR clonotypes from each sample and showing the relationship of the top V(D)J pairing frequencies of expanded clonotypes for sample (right) and contacts (left) among five B-cell clusters.
