# Supplementary material for: Remodeling of anti-tumor immunity with antibodies targeting a p53 mutant
Source: J Hematol Oncol. 2024 Jun 18;17:45. doi: 10.1186/s13045-024-01566-1 (PMC11184848; doi:10.1186/s13045-024-01566-1)
Supplement: Supplementary file 2 — Supplementary Material 2 [file 13045_2024_1566_MOESM2_ESM.pdf]

Marker genes for cell subsets

Cell type	Marker
Cd45 leukocytes	Ptprc
BCs (B cells)	Cd19, Cd22, Cd79a, Cd79b
B_like (B like cells)	Cd79b,Ly6a
IFIT^{+}_BCs	Cd79b, Ifit
Rest_BCs	Cd79b, Hspa1b, Hspa1a
TCs (T cells)	Cd3d, Cd3e, Cd3g, Trac
CD4_TCs (CD4^{+}T cells)	Cd3e, Cd4
CD4_Naive	Cd4,Cd44, Sell
CD4_Cytotoxic	Cd4, Gzmk, Gzmb
Treg (Regulatory T cells)	Cd4, Foxp3
Th1 (T helper 1 cells)	Cd4, Tbx21, Cxcr6
Th17 (T helper 17 cells)	Cd4, Il17a, Rora
Tfh (T follicular helper cells)	Cd4, Cd40l, Tox2, Il4
CD8_TCs (CD8^{+}T cells)	Cd3e, Cd8a, Cd8b1
CD8_Naive	Cd8b1, Sell, Ly6c2, Tcf7
CD8_Tem (Memory-like CD8^{+}T cells)	Cd8b1, Il7r, Icos
CD8_Tpex (Progenitor exhausted CD8^{+} T cells)	Cd8b1, Mki67, Top2a, Ccnb2
CD8_Tex (Exhausted CD8^{+} T cells)	Cd8a,Pdcd1, Lag3, Ifng, Pf1
NKT	Cd3e, Ncr1, Klrb1c, Nkg7
NK_like	Ncr1, Klrb1c, Nkg7, Cd3e, Cd79b
NK	Ncr1, Klrb1c, Nkg7
Cytotoxic_NK	Nkg7, Ifng, Gzma, Cxcr3
Xcl^{+}_NK	Ncr1, Klrb1c, Nkg7, Xcl
Dut^{+}_NK	Ncr1, Klrb1c, Nkg7, Dut
Ifng^{+}_NK	Ncr1, Klrb1c, Nkg7,Ifng
Cd74^{+}_NK_like	Ncr1, Klrb1c, Nkg7, Cd74
Cd3d^{+}_NK_like	Ncr1, Klrb1c, Nkg7, Cd3d
Exhusted_NK_like	Ncr1, Klrb1c, Nkg7, Prf1, Pdcd1,
Actived_NK_like	Ncr1, Klrb1c, Nkg7, Ifng, Gzma, Cxcr3
DCs	Itgam, Itgax
Non TIICs (Tumor-infiltrating immune cells)	Ptprc (Negative)
Neutrophils	S100a8, S100a9;
Macro (Macrophages)	Itgam, Csf1r, Adgre1, Fcgr2b, Fcgr3
Monocytes	Itgam, Csf1r, Gsr, Ly6c2
